# Supplementary figures and images for: An Excess of Gene Expression Divergence on the X Chromosome in Drosophila Embryos: Implications for the Faster-X Hypothesis
Source: PLoS Genet. 2012 Dec 27;8(12):e1003200. doi: 10.1371/journal.pgen.1003200 (PMC3531489; doi:10.1371/journal.pgen.1003200)

## Embryos

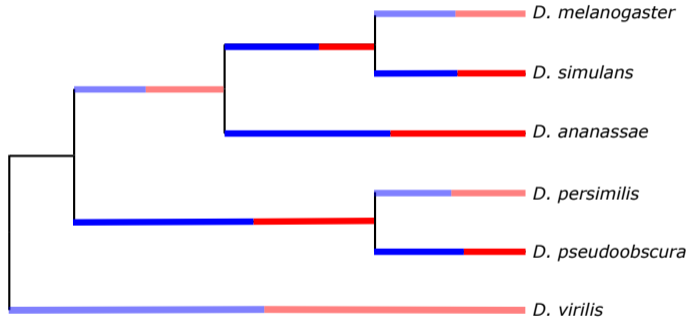

## Adults

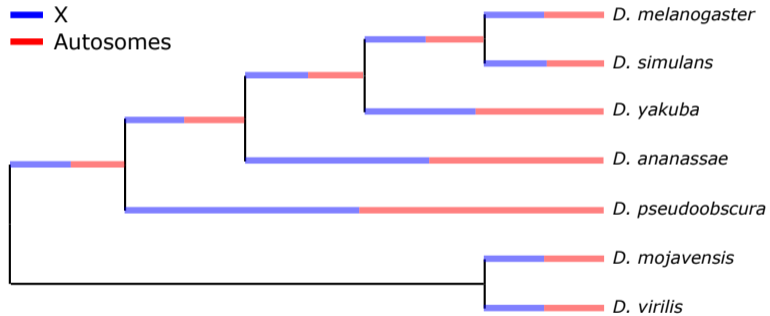

Supplement: Figure S1 — Phylogenies of the species analyzed with the relative mean lengths of each branch for genes on the X vs genes on the autosomes depicted in blue and red respectively. Bold branches are significantly longer for genes on the X chromosome based on 10,000 bootstrap replicates at the 5% level. (PDF) [file pgen.1003200.s001.pdf]

## Males

### Male-biased

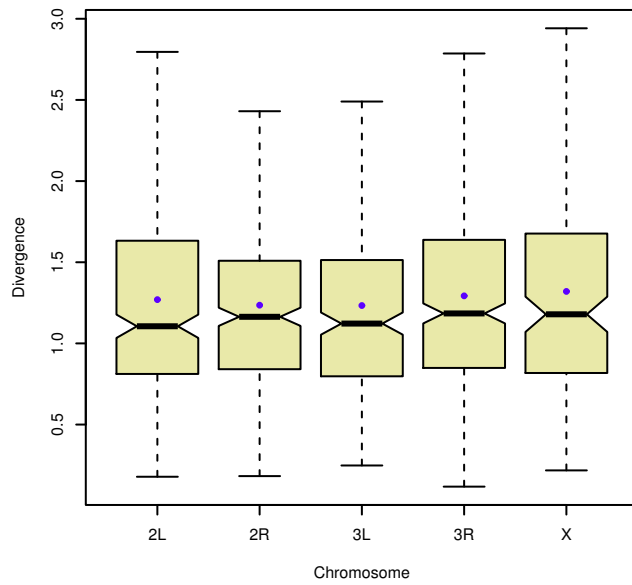

### Female-biased

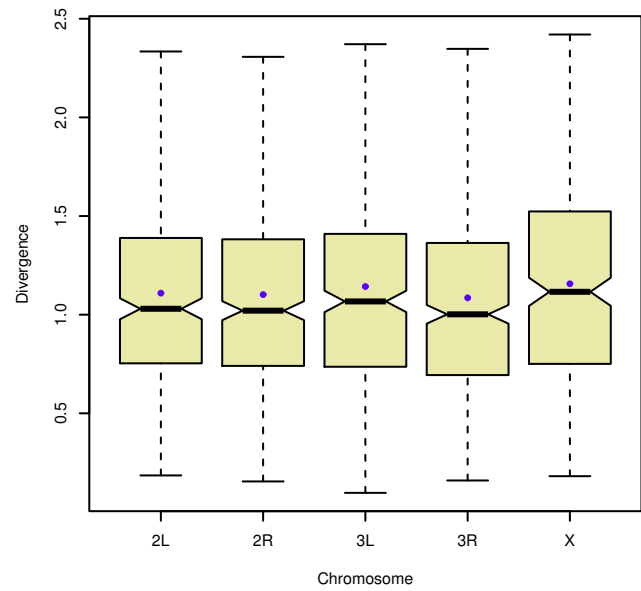

## Females

### Female-biased

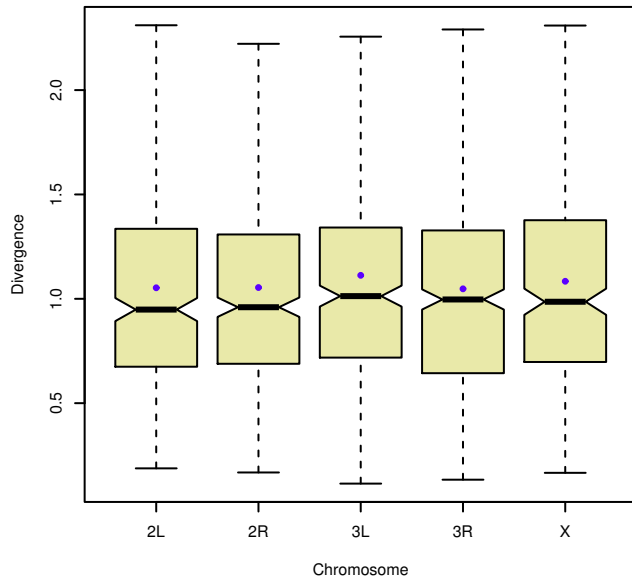

### Male-biased

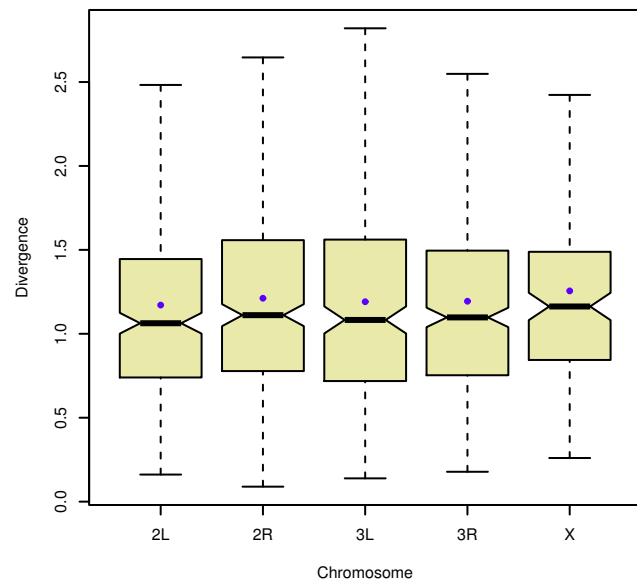

Supplement: Figure S2 — Divergence of gene expression across chromosomes in both adult males and females for genes with sex-biased expression patterns. (PDF) [file pgen.1003200.s002.pdf]

A

Embryos

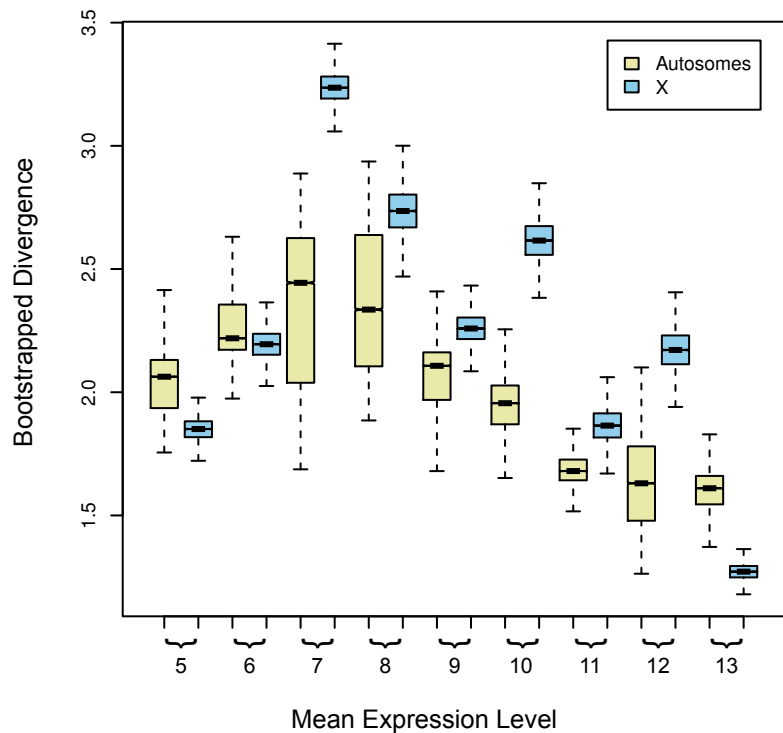

B

Adults

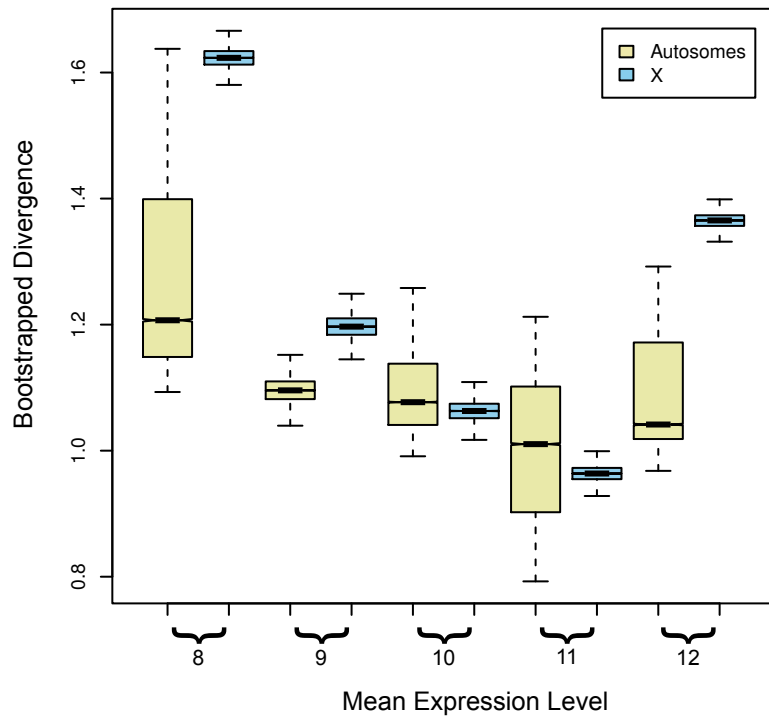

Supplement: Figure S3 — Embryonic expression divergence on the X is not driven by extreme expression levels. Bootstrapped divergence measures generated by resampling genes according to their expression levels. Genes were resampled per chromosome using 10,000 bootstrap replicates for both embryos, A, and adults, B. There are more expression levels sampled for embryos because they have a broader gene expression level distribution than the adults. (PDF) [file pgen.1003200.s003.pdf]

mel

per

pse

sim

vir

ana

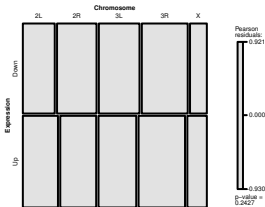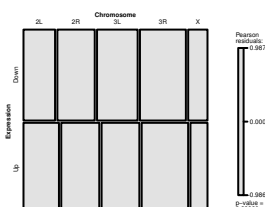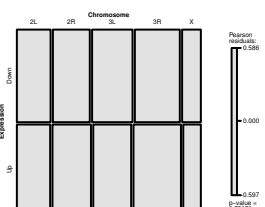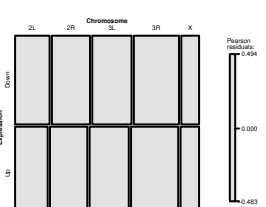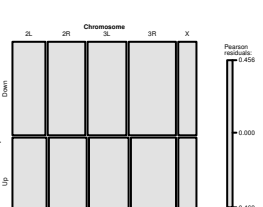

mel

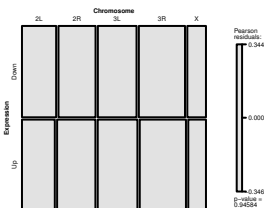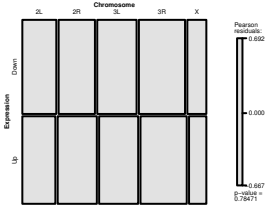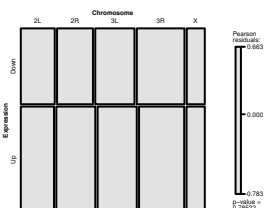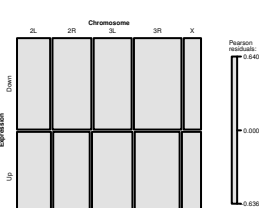

per

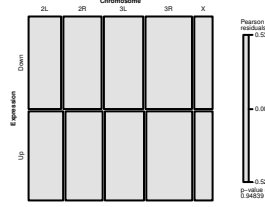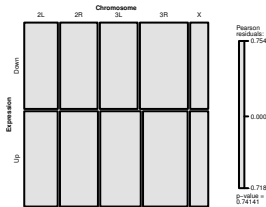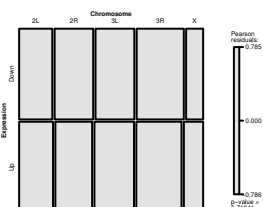

pse

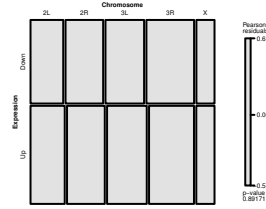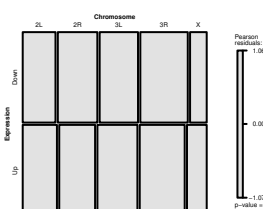

sim

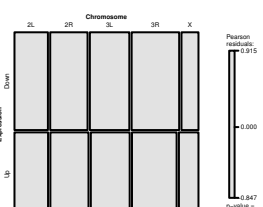

Supplement: Figure S4 — Mosaic plots for all pair-wise species comparisons of normalized gene expression categorised as up or down relative to one of the species. Mosaic plots visualize categorical data (contingency table) using rectangles that are proportional to the number of counts in each row-column combination, and highlight in red variable combinations that have less than expected numbers and in blue those that have more than expected based on Pearson residuals [85]. -values are based on Chi-squared tests, which test whether the two main variables, Expression and Chromosome, are independent. (PDF) [file pgen.1003200.s004.pdf]

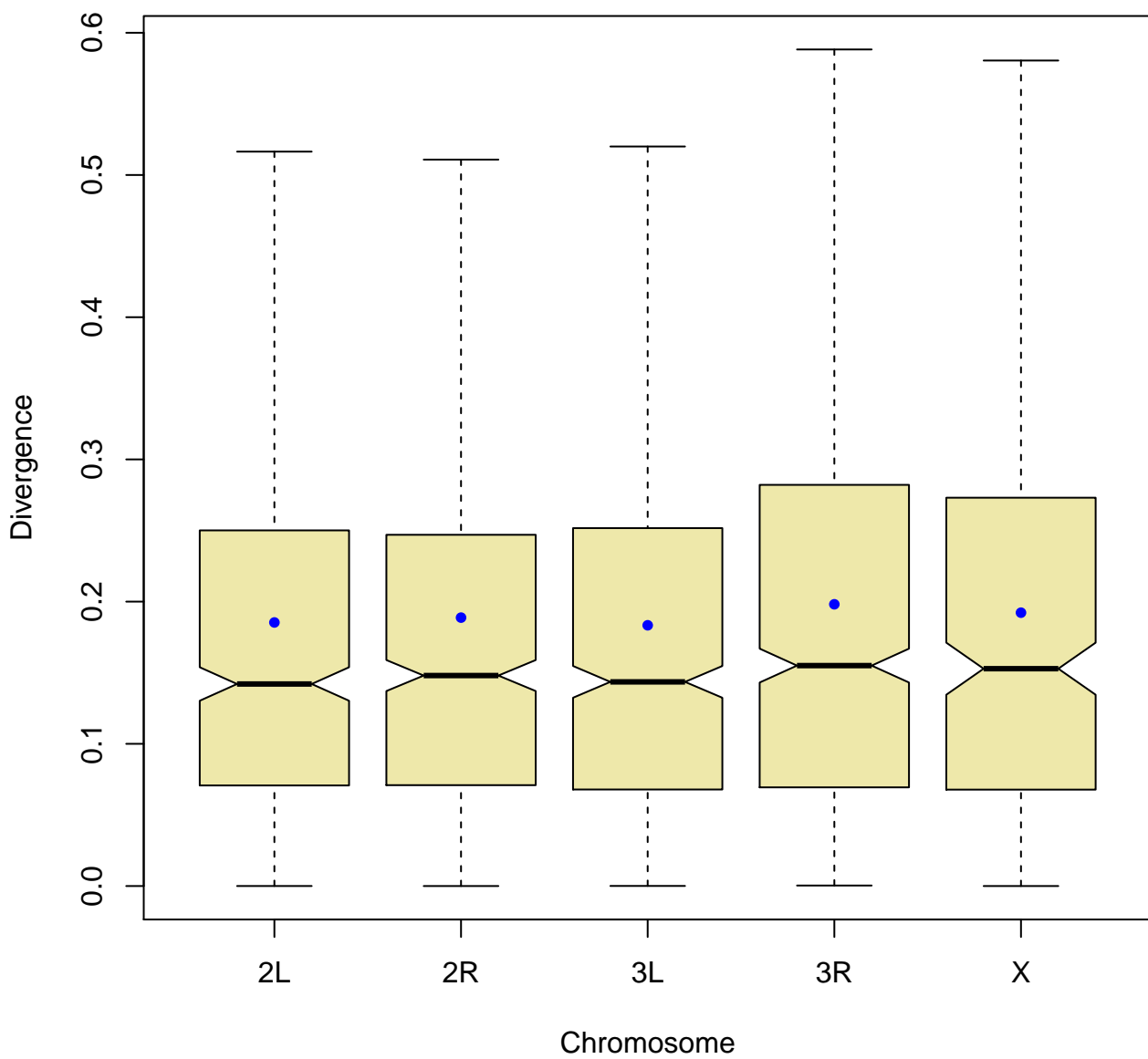

Supplement: Figure S5 — The lengths of the summed terminal branches leading to D. persimilis and D. pseudoobscura as a fraction of the total branch length for Drosophila embryos. (PDF) [file pgen.1003200.s005.pdf]

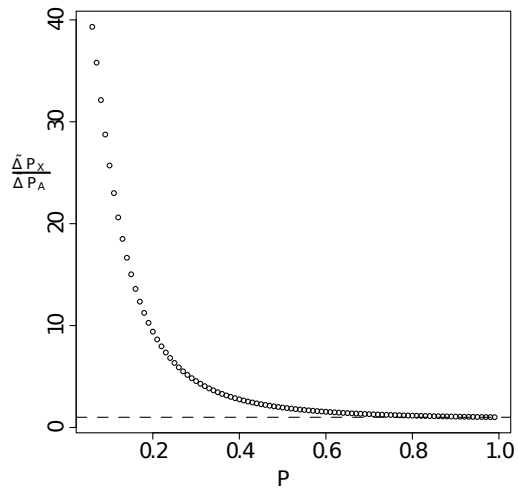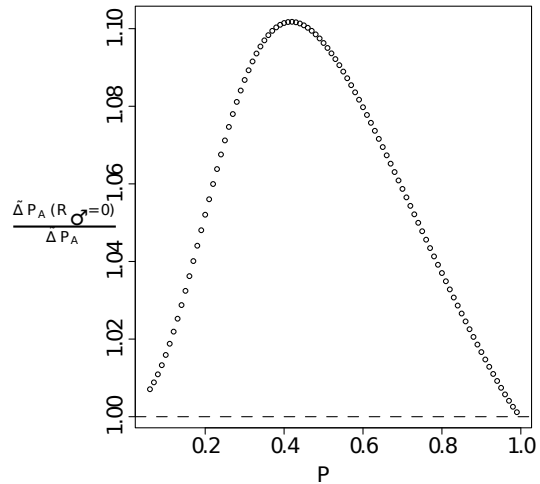

Supplement: Figure S6 — Selection gradient ratios when there is no recombination between homologous pairs of male autosomes. The left panel shows the ratio when both loci are X-linked versus both loci being linked on the same autosome but with no male recombination. The right panel shows the ratio for autosomes when there is no recombination in males versus the case when there is. Parameter values: recombination rates, , are equal to 0.5 (free recombination) and the dominance coefficient, , is 0.01. The dashed line indicates a ratio of 1. (PDF) [file pgen.1003200.s006.pdf]

Mel-sim male-bias in  
Males

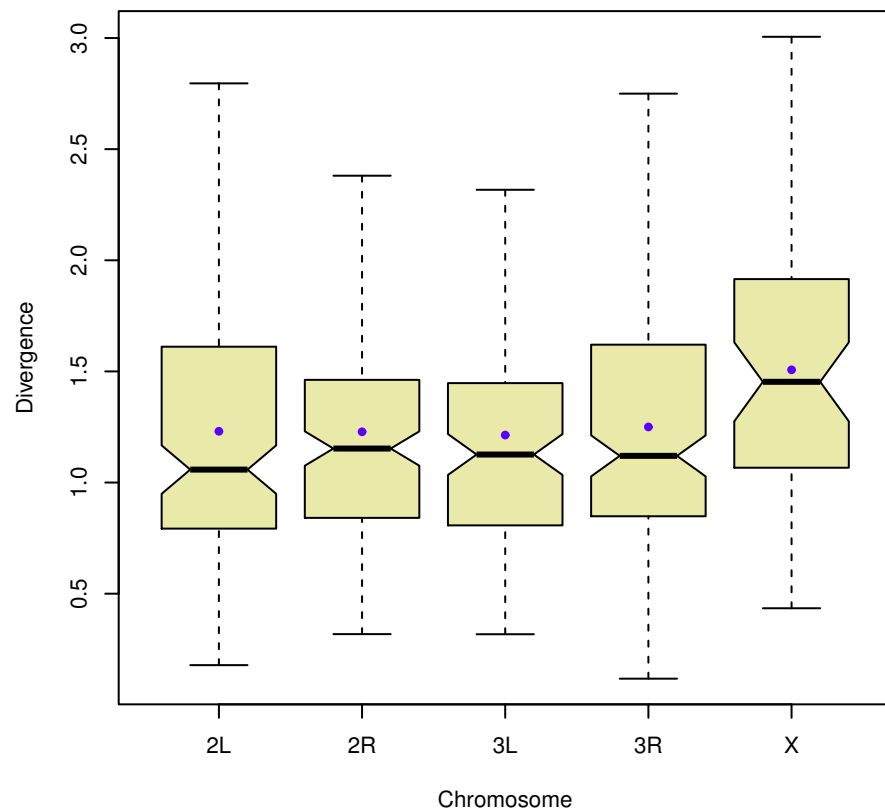

Mel-sim male-bias in  
Females

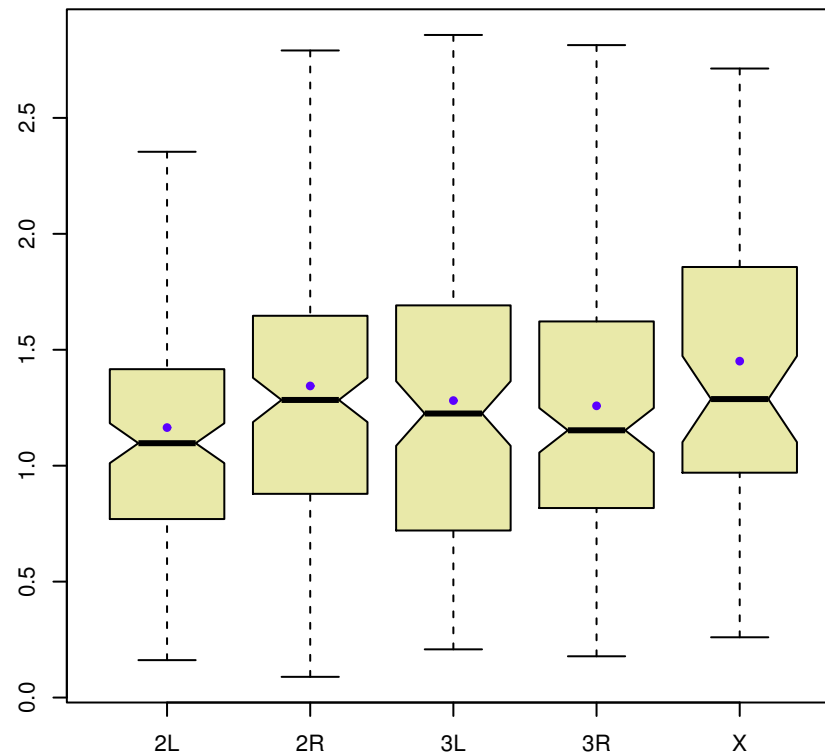

Supplement: Figure S7 — Divergence of gene expression across chromosomes in both adult males and females for 656 genes with male-biased expression in either D. melanogaster or D. simulans. (PDF) [file pgen.1003200.s007.pdf]

# Correlation (Spearman's $\rho$ )

Males

Females

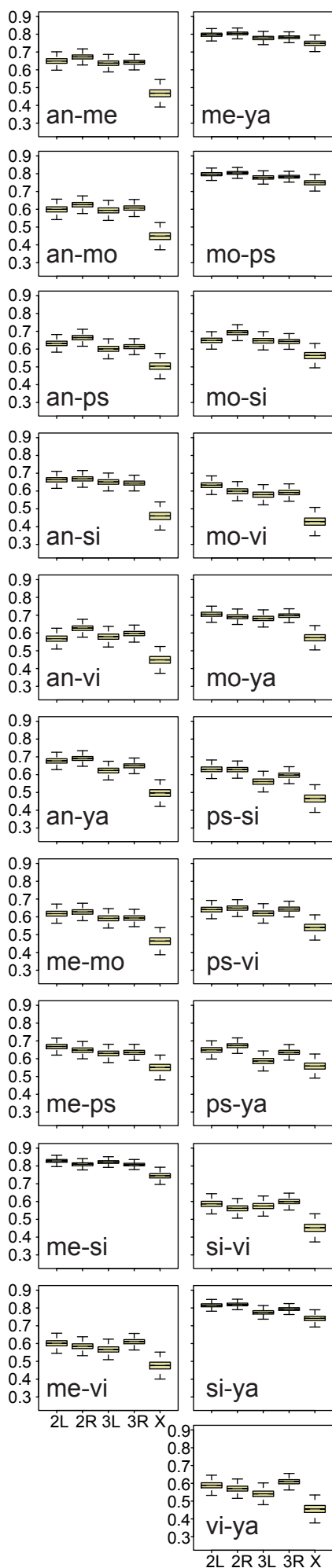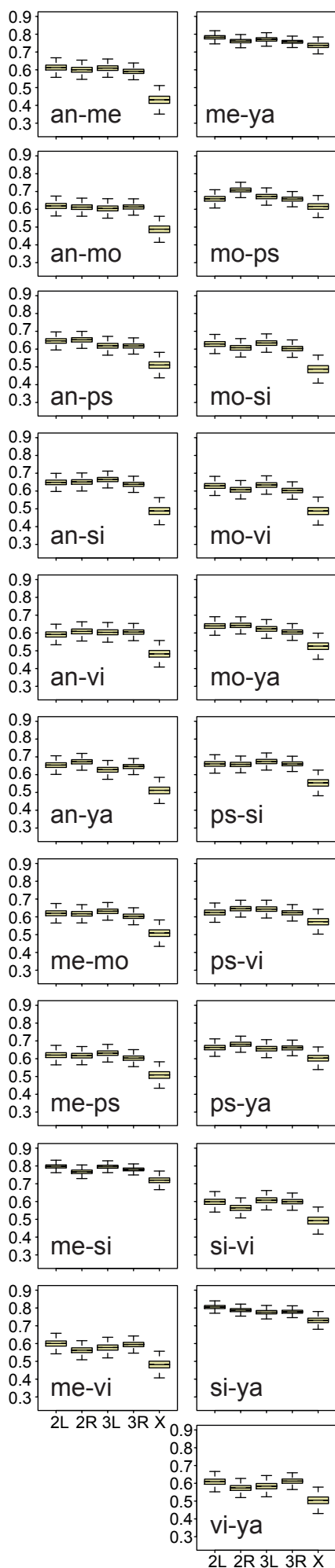

Supplement: Figure S8 — Bootstrapped (10,000 replicates) Spearman's correlation coefficients for adult males and females for all pair-wise species comparisons. (PDF) [file pgen.1003200.s008.pdf]

Distance (Canberra)

Males

Females

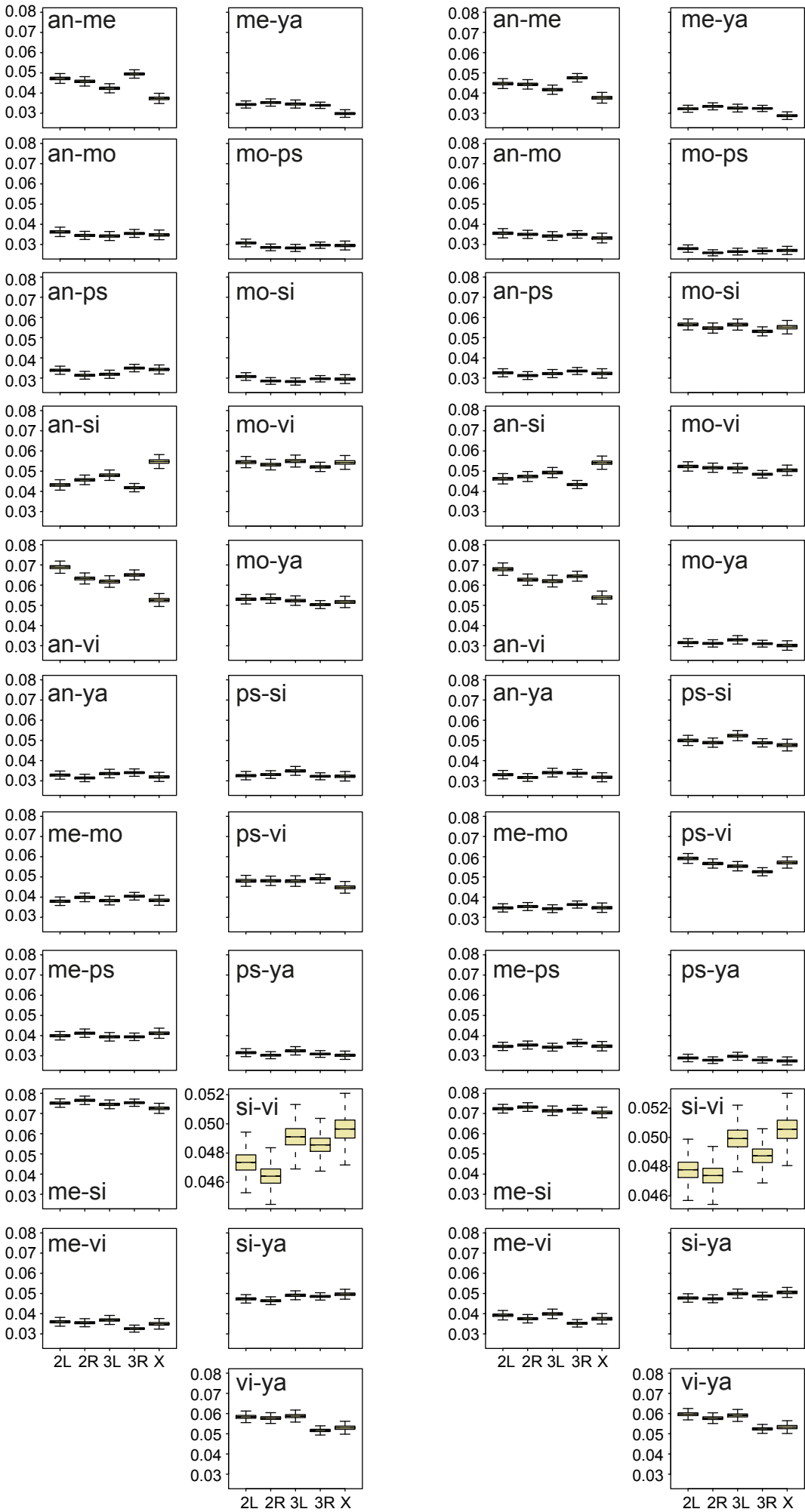

Supplement: Figure S9 — Bootstrapped (10,000 replicates) Mean Canberra distances for adult males and females for all pair-wise species comparisons. (PDF) [file pgen.1003200.s009.pdf]

Embryo

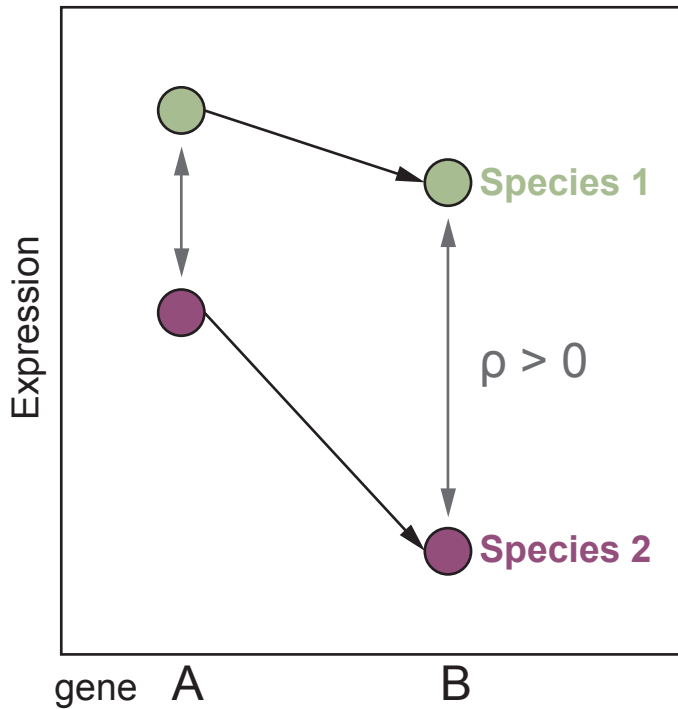

Adult

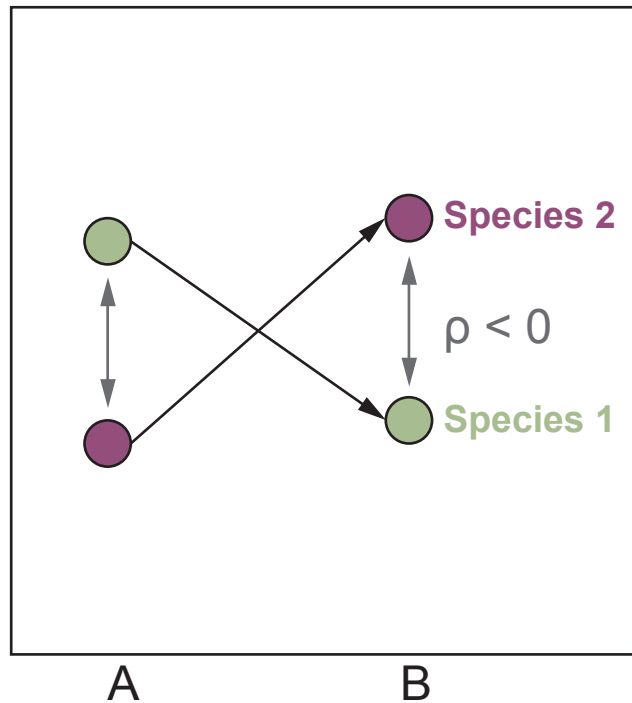

Supplement: Figure S10 — A schematic depicting gene expression in two genes showing why Spearman's would produce a positive correlation despite large differences in expression level and a negative correlation when expression co-ordination between genes is diminished regardless of how much absolute gene expression levels have changed. (PDF) [file pgen.1003200.s010.pdf]

Bootstrapped Correlation of Gene Expression

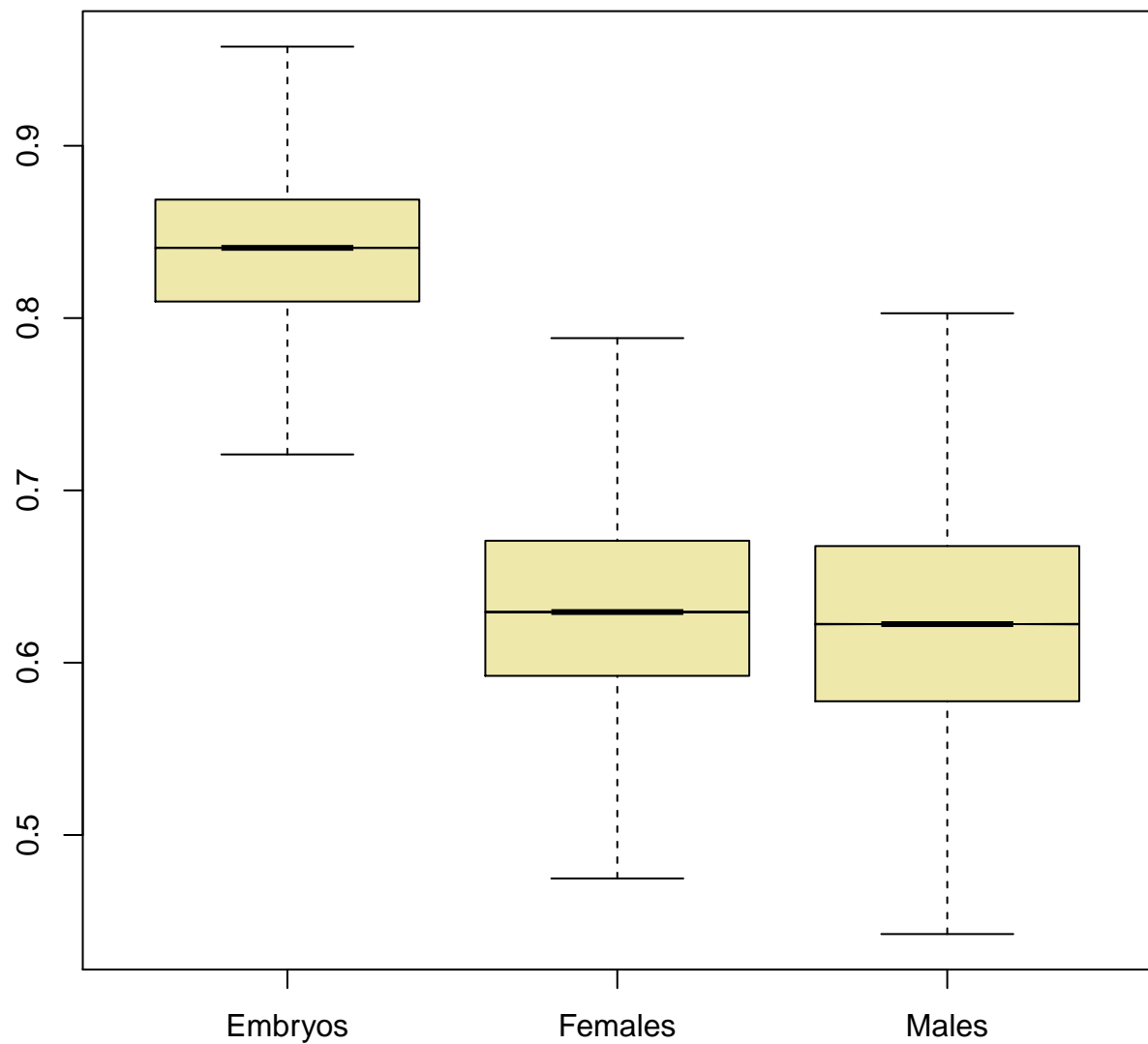

Supplement: Figure S11 — All bootstrapped Spearman's correlations across all chromosomes for embryos and adult males and females. (PDF) [file pgen.1003200.s011.pdf]

t1

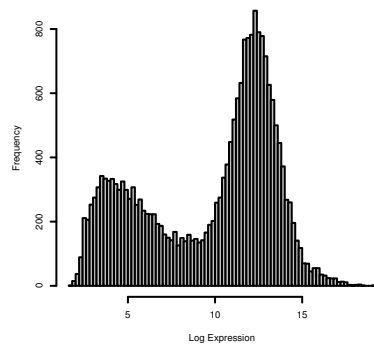

t2

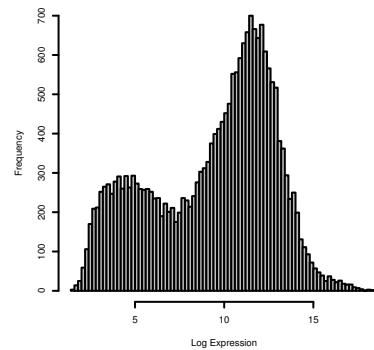

t3

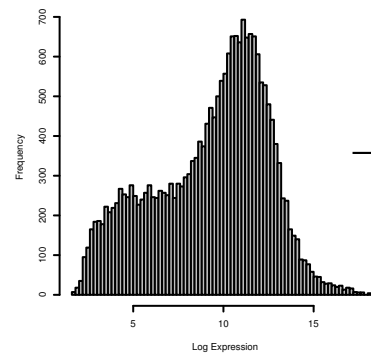

t4

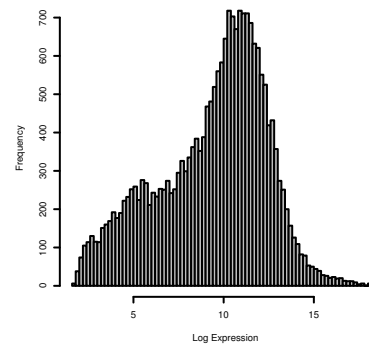

t5

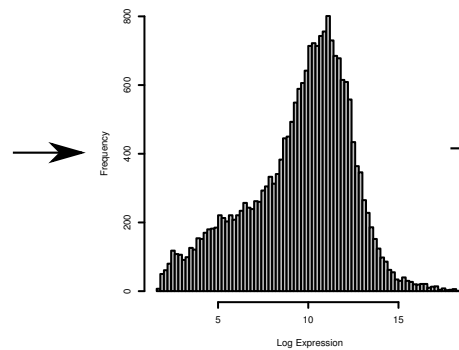

t6

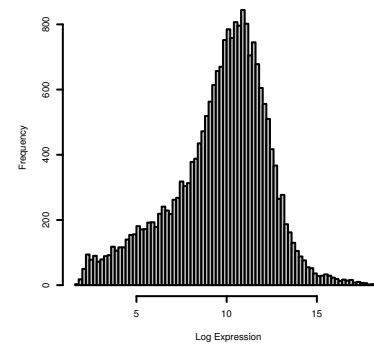

t7

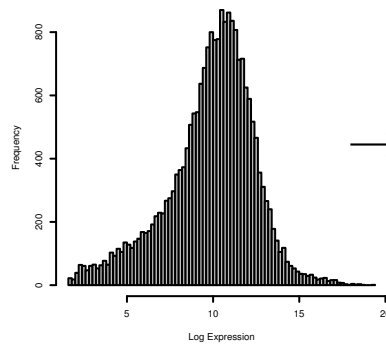

t8

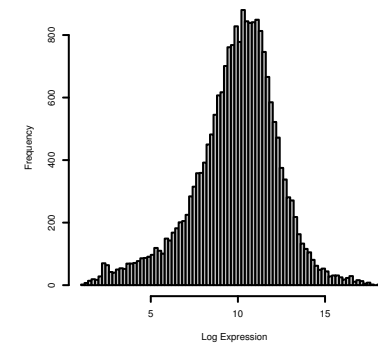

Supplement: Figure S12 — The distribution of gene expression levels during embryogenesis of D. melanogaster showing that an initially bimodal distribution, where the lower mode represents unexpressed zygotic genes, becomes a unimodal distribution through time as the zygotic genome is activated. (PDF) [file pgen.1003200.s012.pdf]

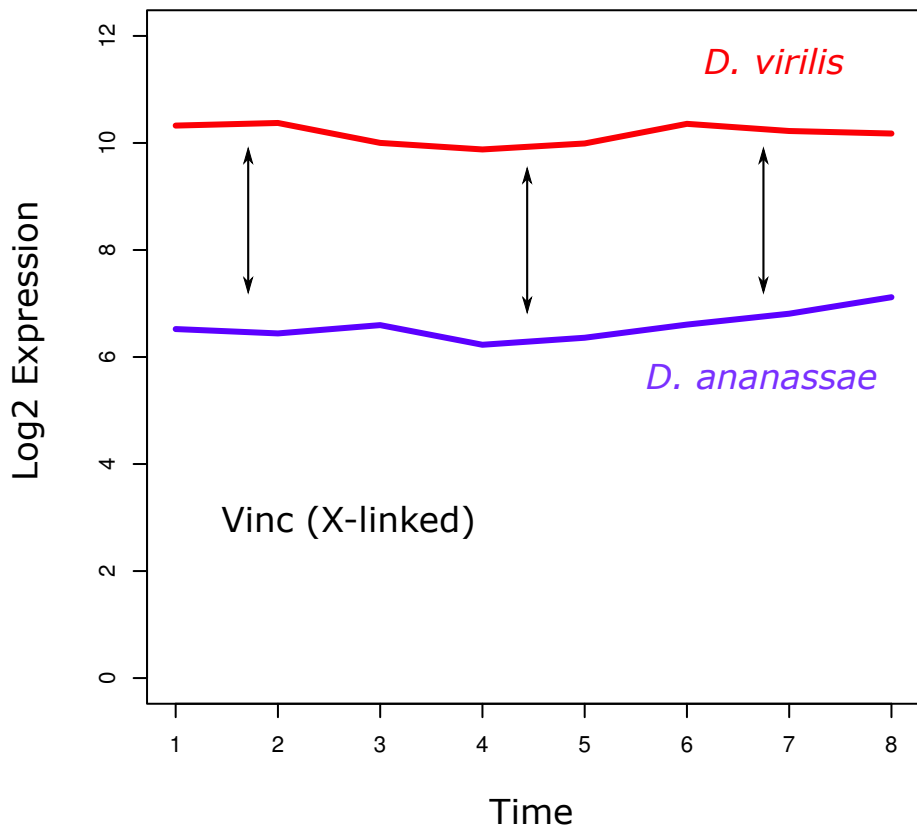

Supplement: Figure S13 — Log2 gene expression time course for the X-linked gene Vinculin (Vinc) for D. ananassae and D. virilis showing divergence across the whole time course. (PDF) [file pgen.1003200.s013.pdf]

## Embryos

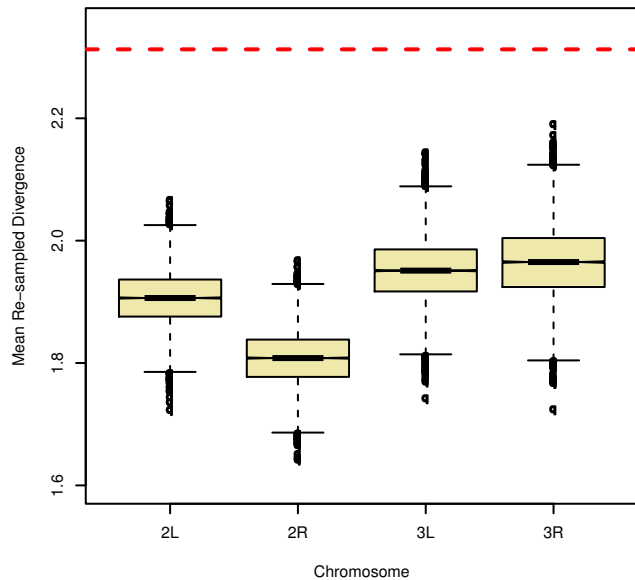

Supplement: Figure S14 — The distributions of resampled mean divergences for each autosome with the mean of the X chromosome indicated by a dashed red line for embryos and adults. Autosomal genes were resampled so that they matched the number of genes on the X chromosome and in each of 10,000 resamples the mean divergence per chromosome was recorded. (PDF) [file pgen.1003200.s014.pdf]

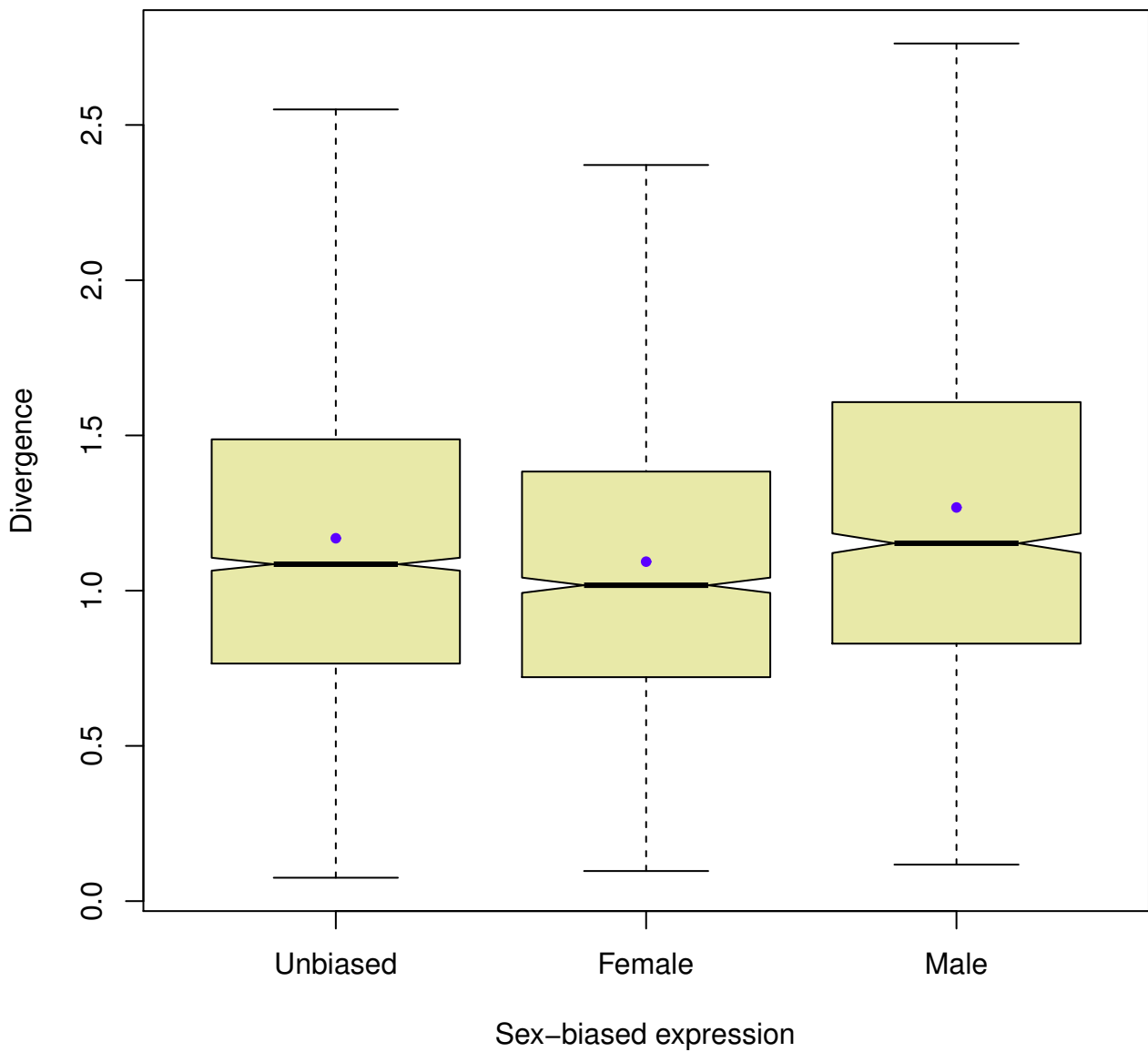

Supplement: Figure S15 — Divergence of gene expression in adult males for genes that show unbiased, male-biased, and female-biased expression patterns. (PDF) [file pgen.1003200.s015.pdf]

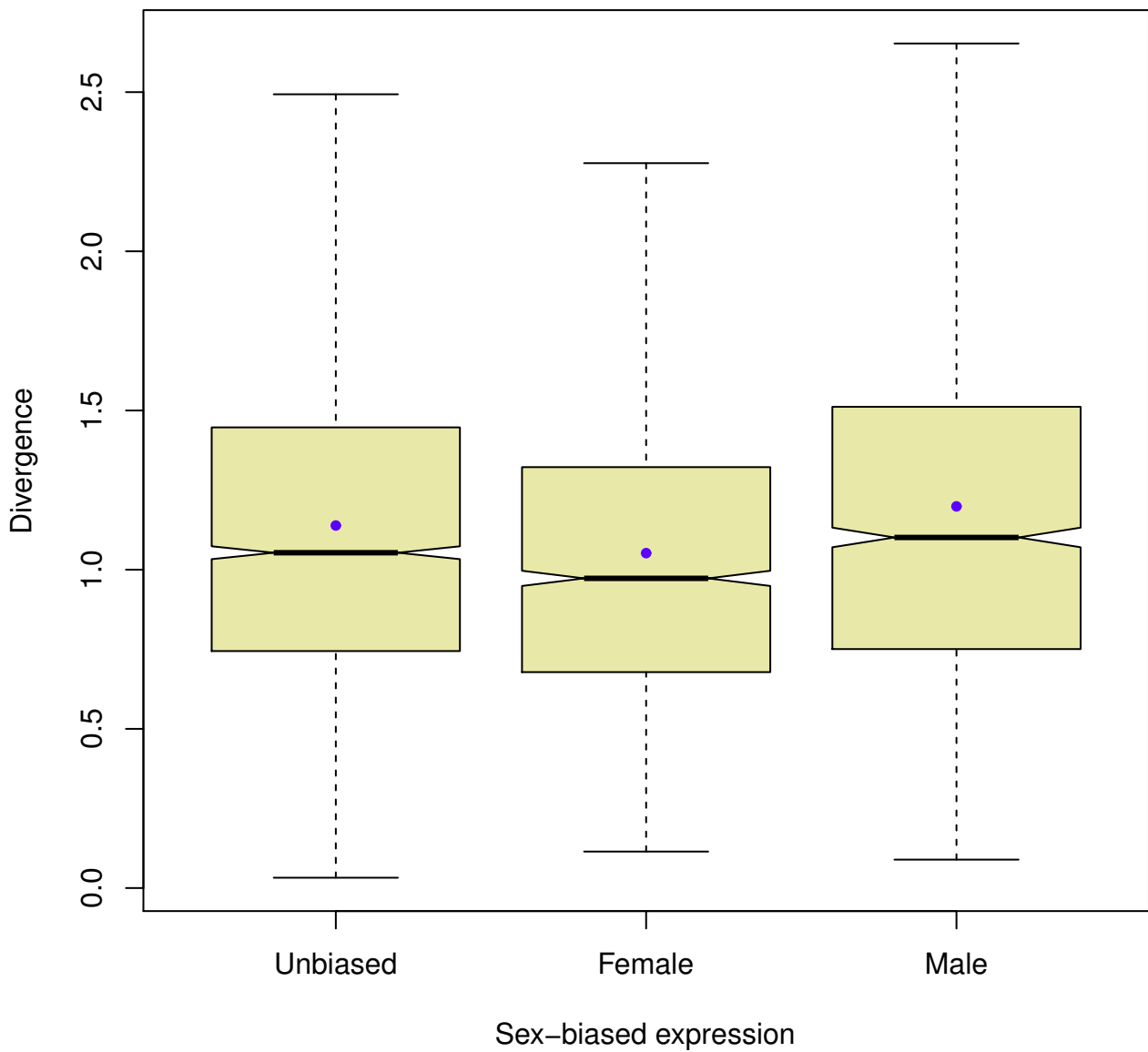

Supplement: Figure S16 — Divergence of gene expression in adult females for genes that show unbiased, male-biased, and female-biased expression patterns. (PDF) [file pgen.1003200.s016.pdf]

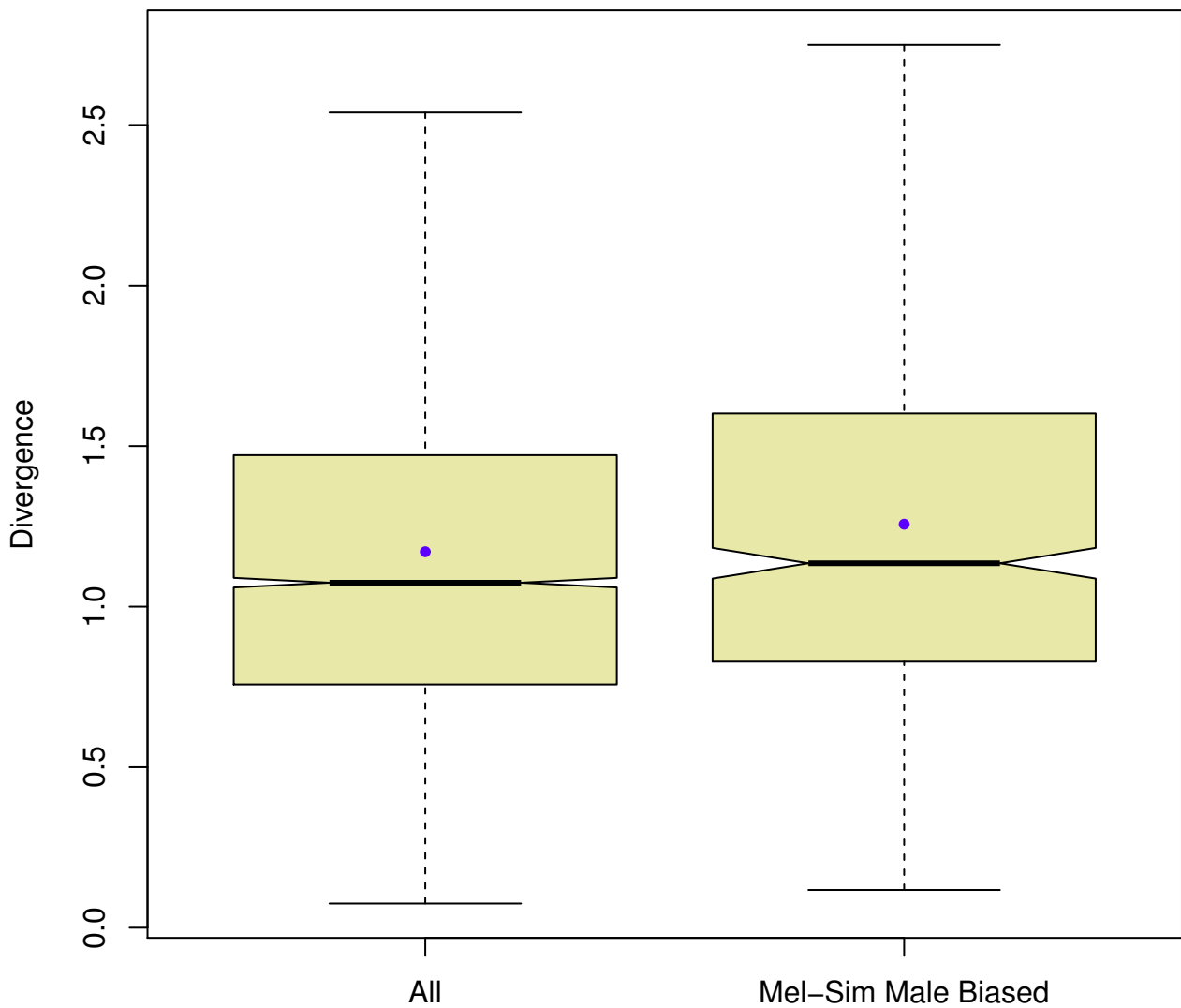

Supplement: Figure S17 — Divergence of gene expression in adult males for 656 genes with male-biased expression in either D. melanogaster or D. simulans relative to all genes in the dataset. (PDF) [file pgen.1003200.s017.pdf]

**Embryos**

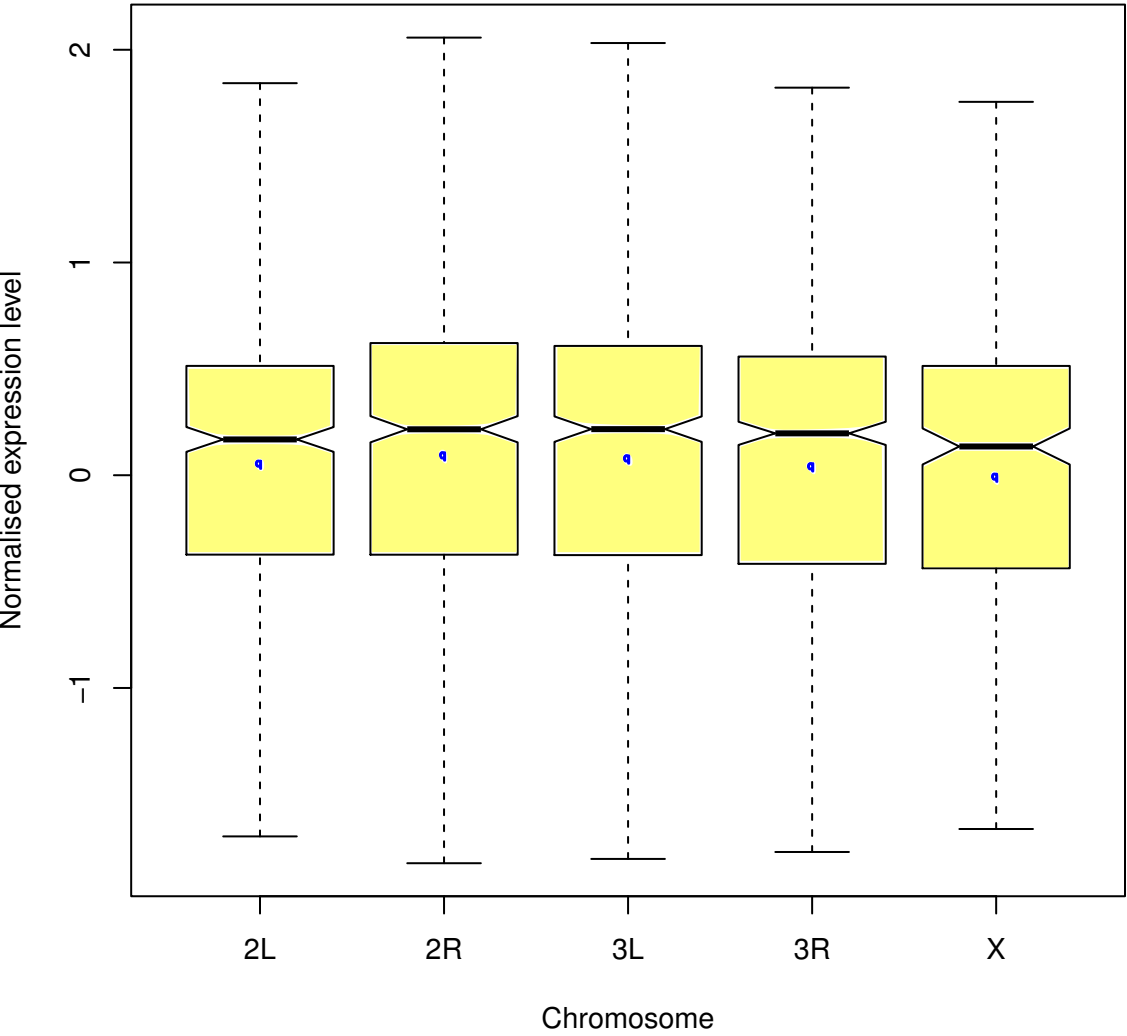

**Adults**

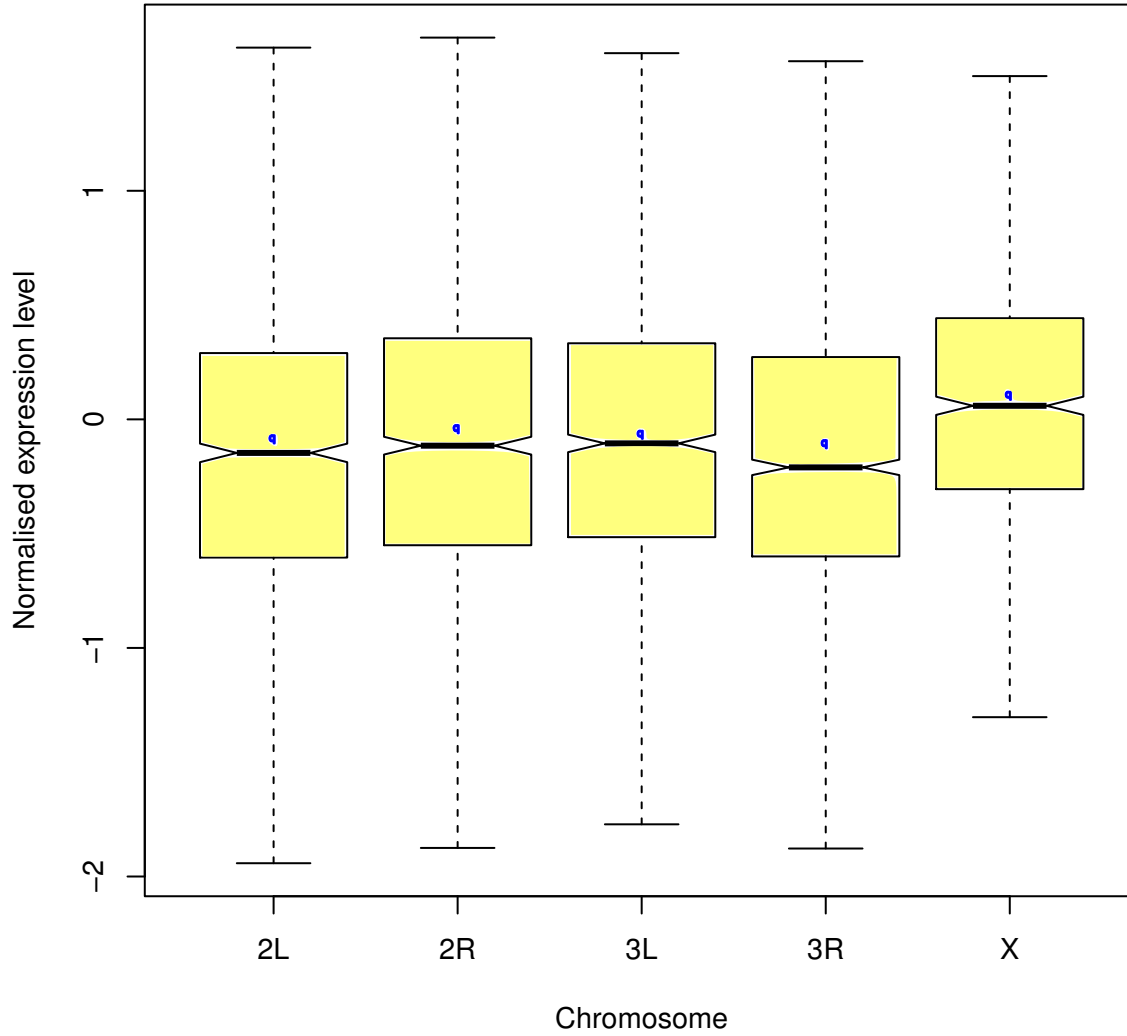

Supplement: Figure S18 — Gene expression level by chromosome for embryos and adults in the Drosophila data sets. Expression level is shown as the deviation of each gene's mean log2 expression level from the global mean. (PDF) [file pgen.1003200.s018.pdf]

### Embryos

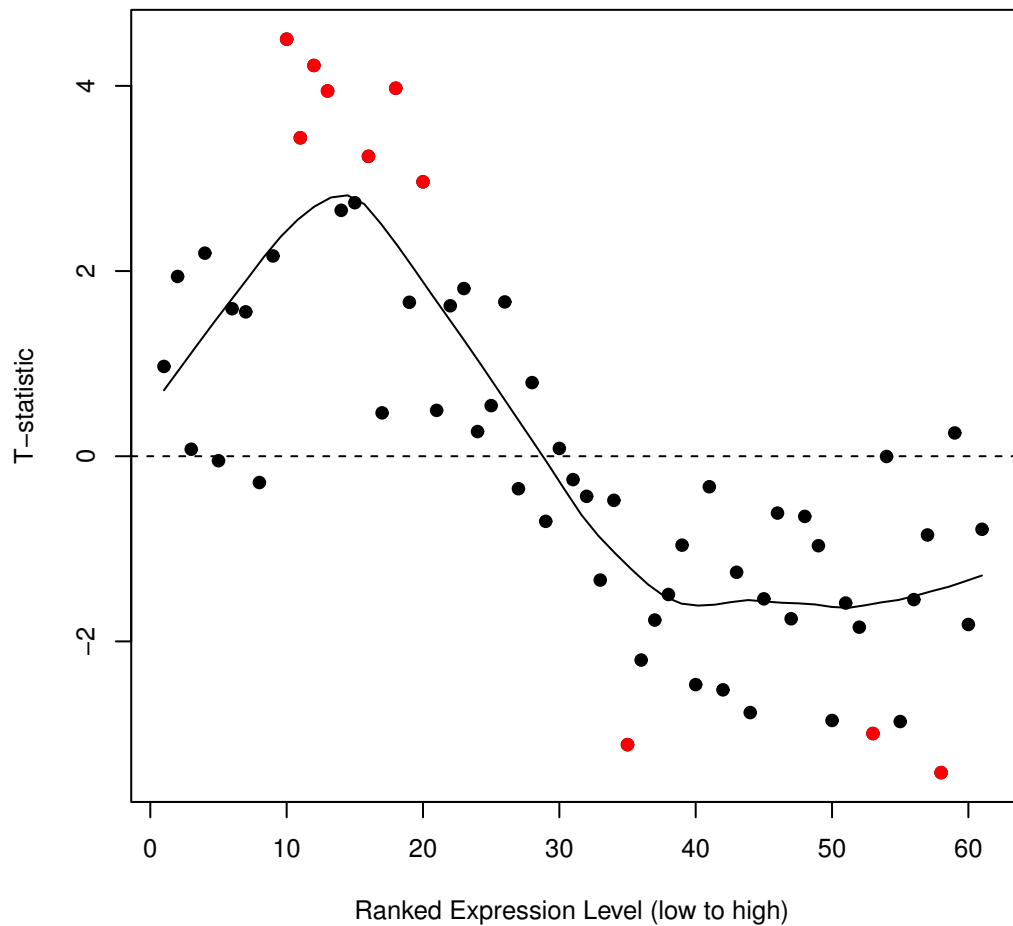

### Adults

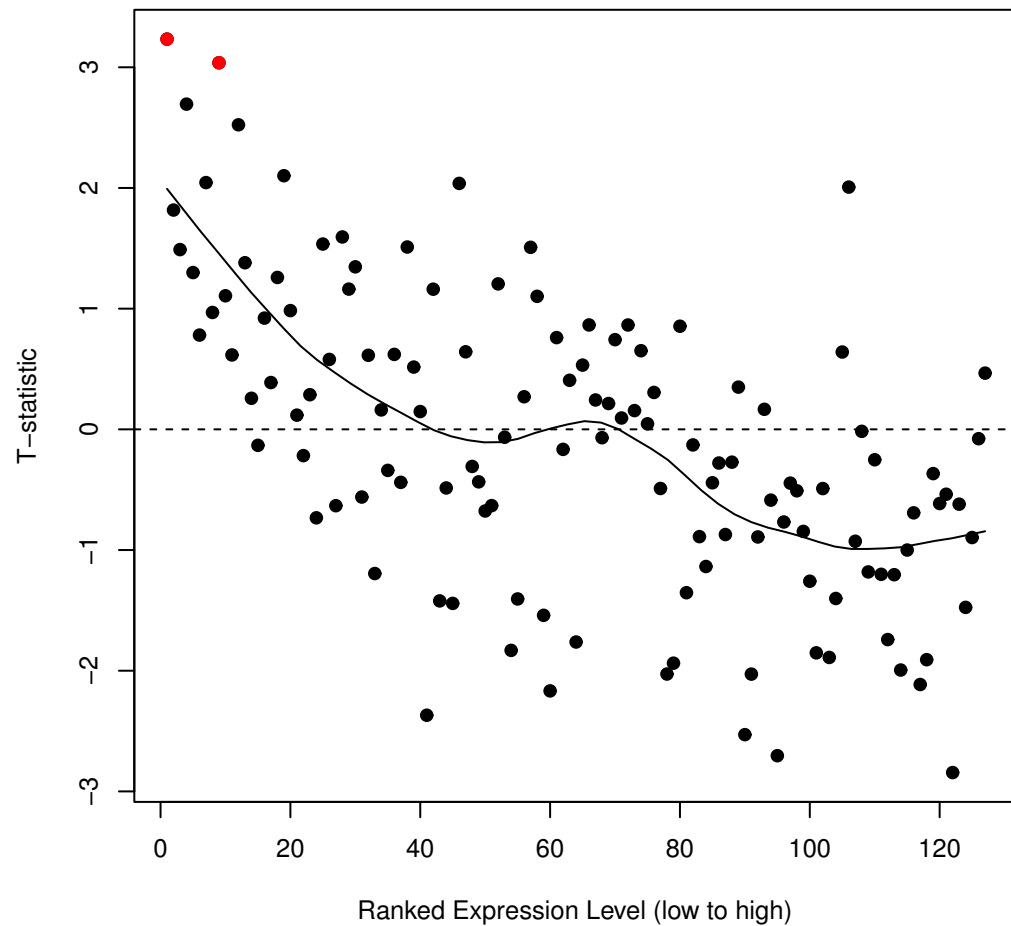

Supplement: Figure S19 — The relationship between expression level and divergence for embryos and adults in the Drosophila data sets. Genes are ranked by expression, from lowest to highest, binned into groups of 50, and their mean divergence deviation from the global mean (log divergence) is shown as a T-statistic, with significant values highlighted in red. A LOESS curve is fitted to the data. (PDF) [file pgen.1003200.s019.pdf]

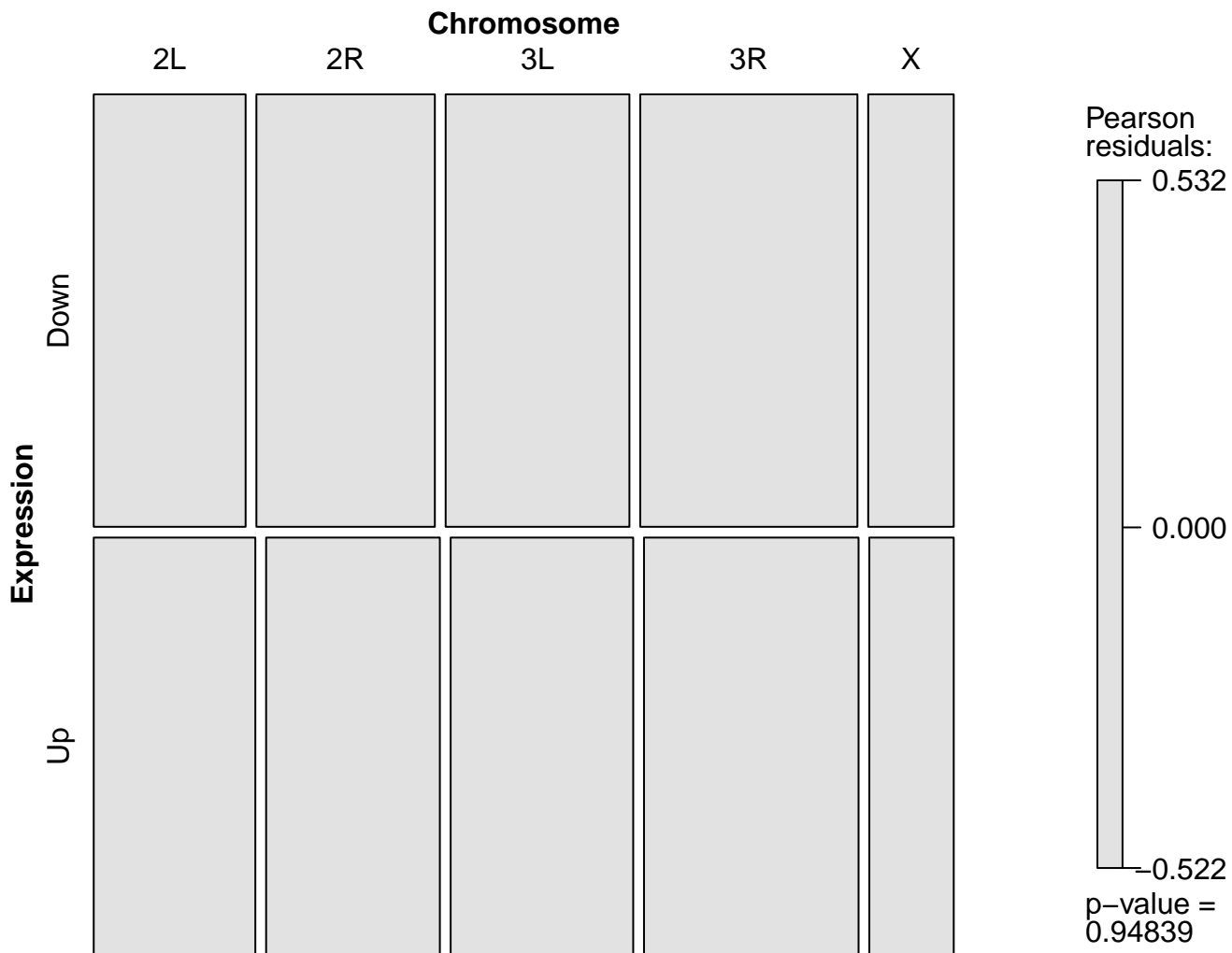

Supplement: Figure S20 — Mosaic plots for the D. persimilis-D. pseudoobscura species comparison of normalized gene expression categorised as up or down relative to one of the species. Mosaic plots visualize categorical data (contingency table) using rectangles that are proportional to the number of counts in each row-column combination, and highlight in red variable combinations that have less than expected numbers and in blue those that have more than expected based on Pearson residuals [85]. -values are based on Chi-squared tests, which test whether the two main variables, Expression and Chromosome, are independent. (PDF) [file pgen.1003200.s020.pdf]

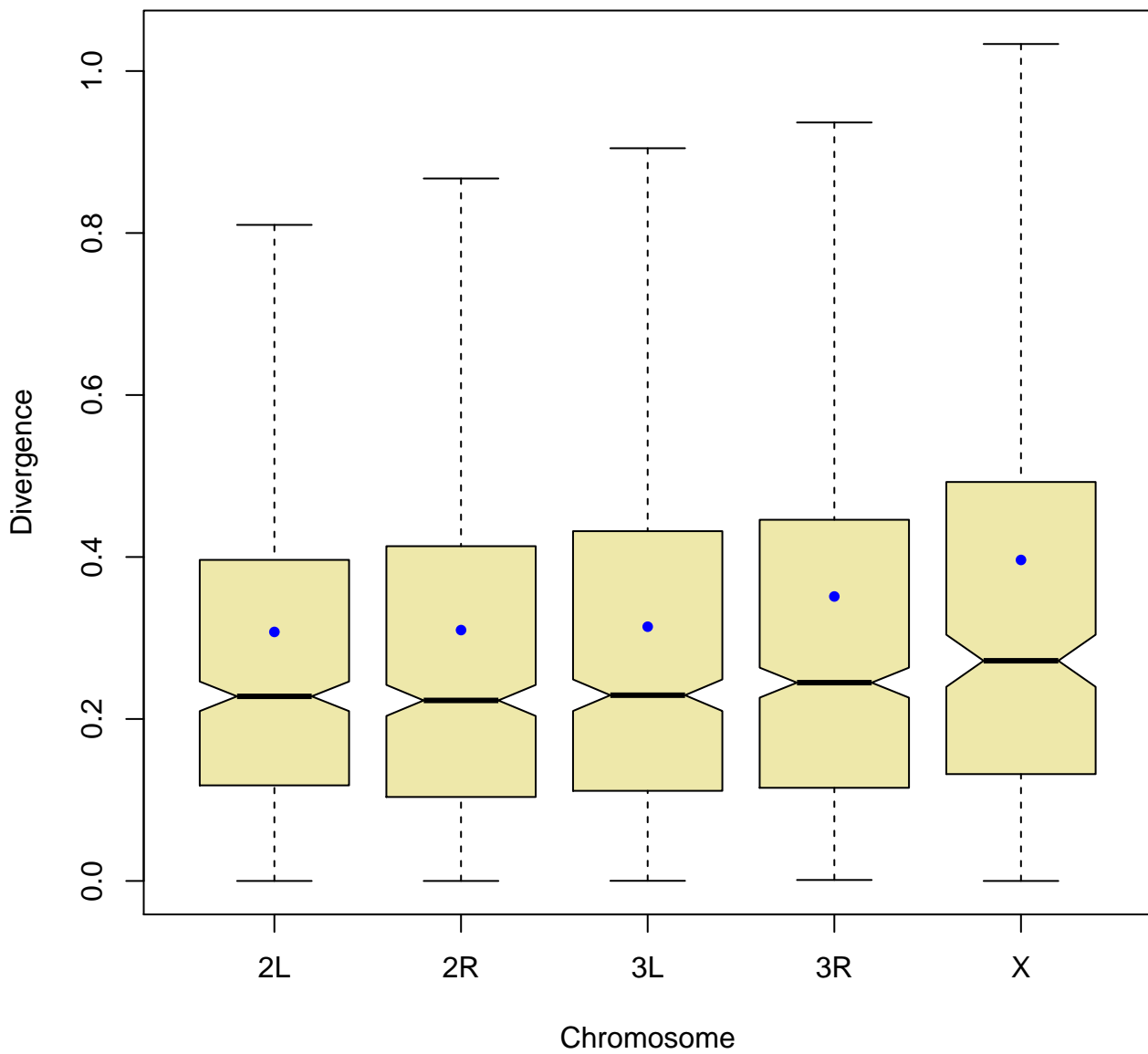

Supplement: Figure S21 — Gene expression divergence per chromosome along the branches leading to D. persimilis and D. pseudoobscura. (PDF) [file pgen.1003200.s021.pdf]

Embryos:

Adult  
(Males):

Adult  
(Females):

Ratio of Chromosomal Divergence

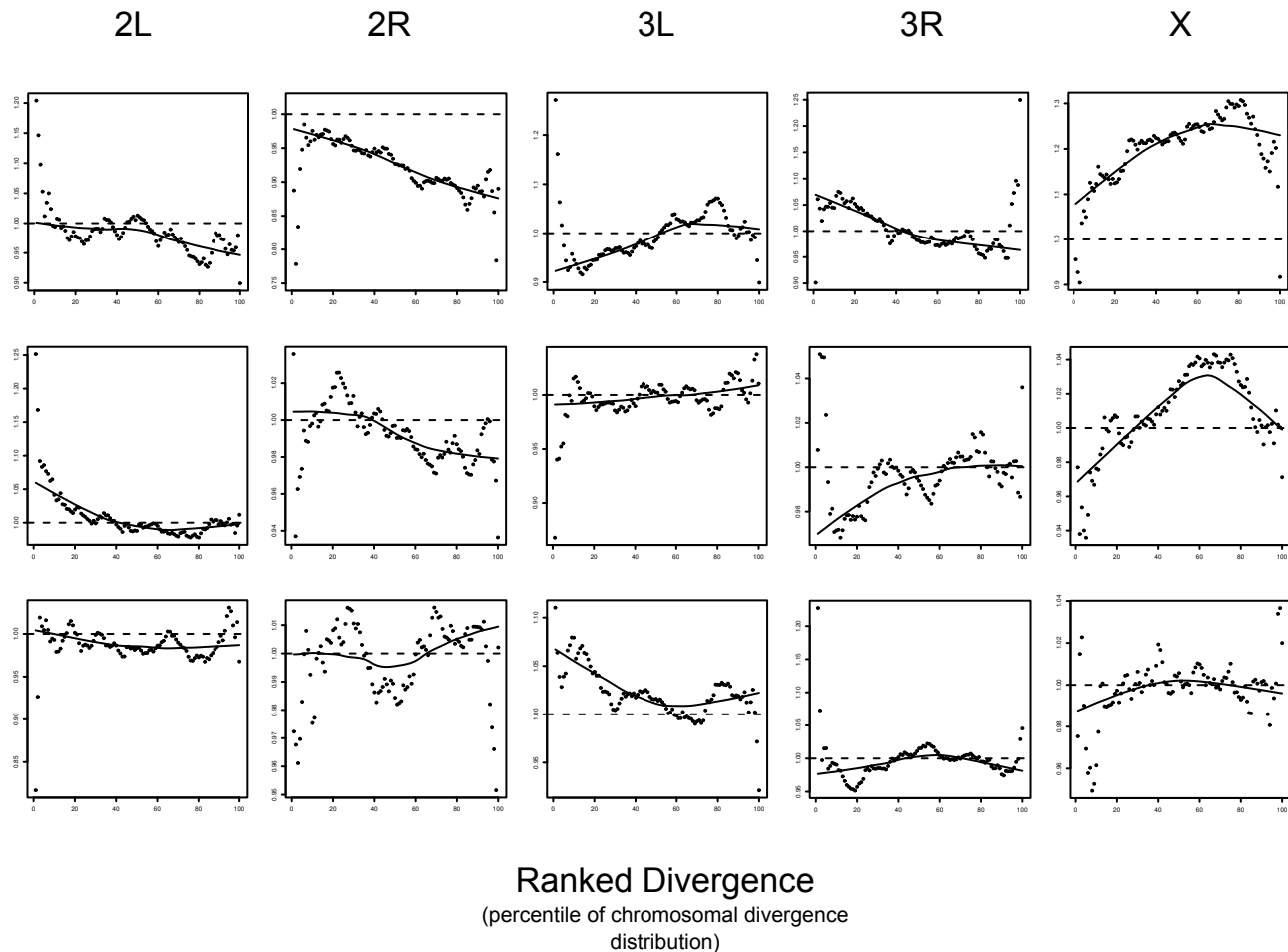

Supplement: Figure S22 — Fast-evolving genes tend to diverge more on the X in embryos and adult males. The mean ratio of chromosomal divergence to divergence in the rest of the genome. Mean divergence is plotted for genes belonging to each percentile of a particular chromosome's divergence distribution (separately for 2L, 2R, etc) relative to genes in the same percentile of the divergence distribution of the the rest of the genome (all other chromosomes). The results show that, for the X chromosome, the excess of X/A divergence is higher for faster-evolving genes in both embryos and adult males. Lines are LOESS fits to the data and dashed lines indicate ratios of 1. (PDF) [file pgen.1003200.s022.pdf]

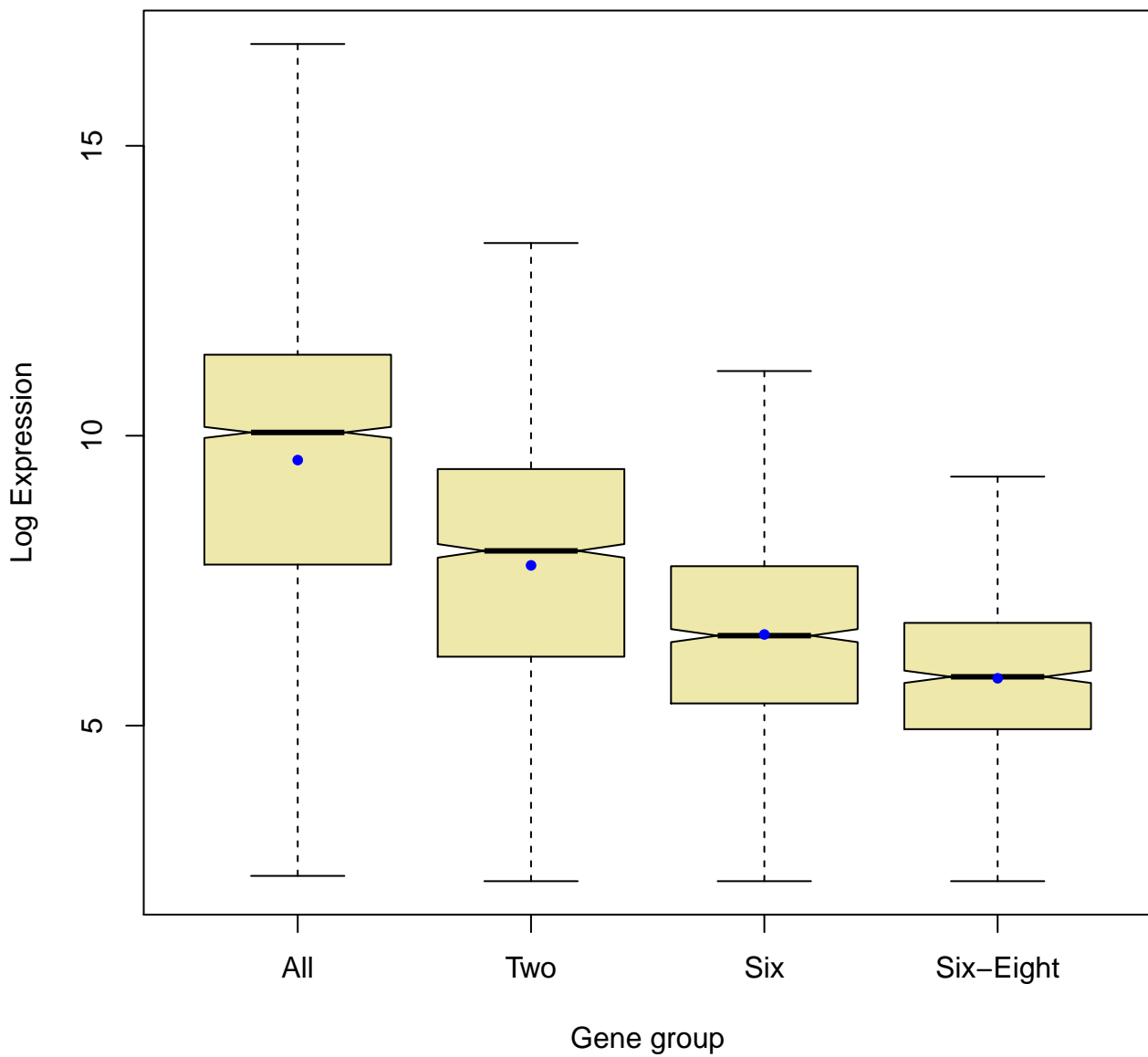

Supplement: Figure S23 — Log expression distributions for gene sets excluded for being non-expressed in at least two species in at least one time point (“Two”), in at least six species in at least one time point (“Six”), and in all species at all time points (“Six-Eight”). See Methods. (PDF) [file pgen.1003200.s023.pdf]

Two

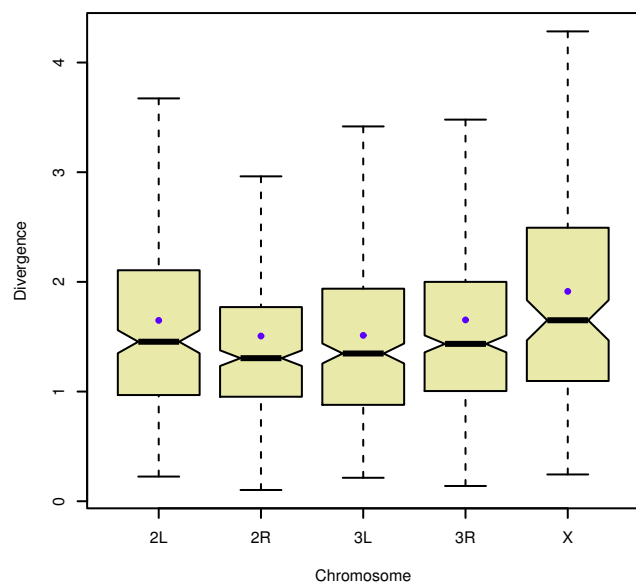

Six

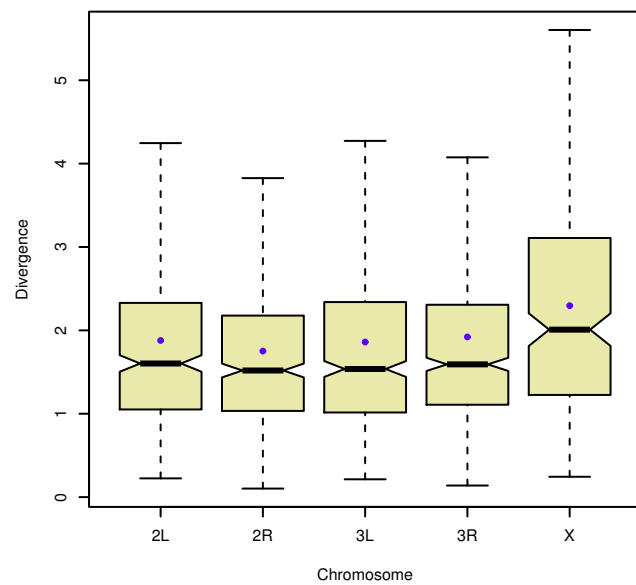

Six-Eight

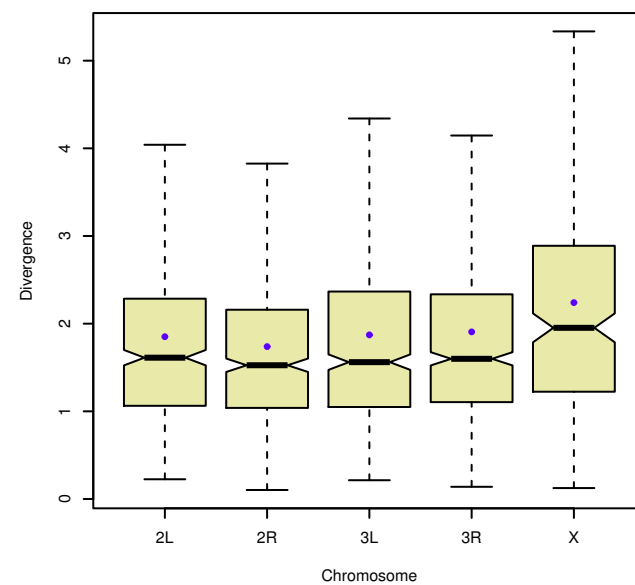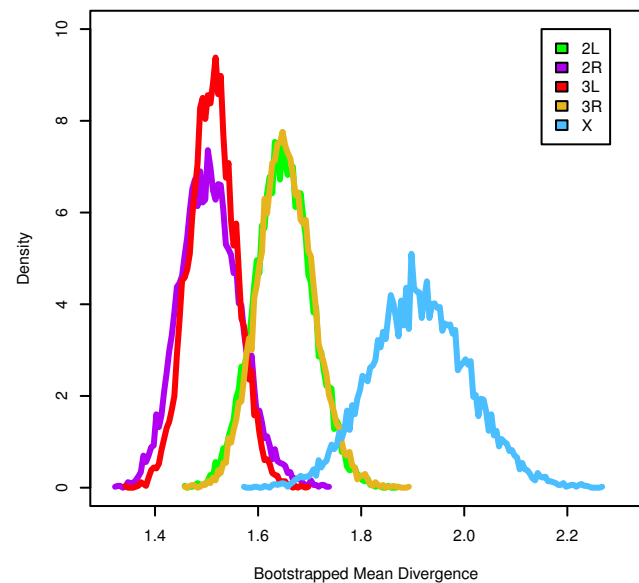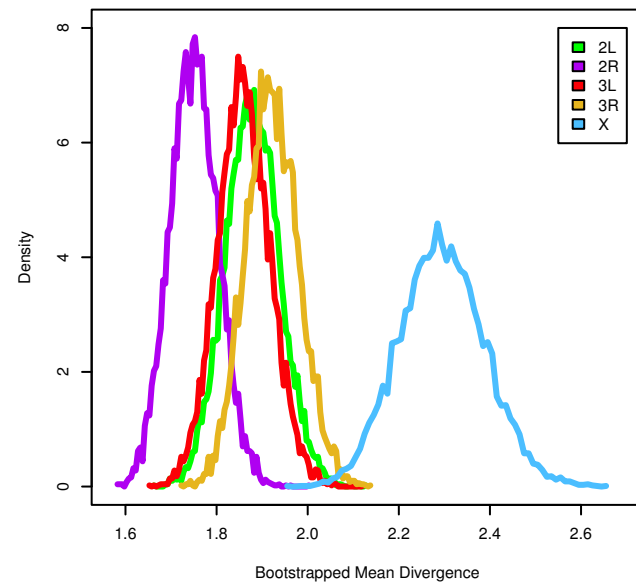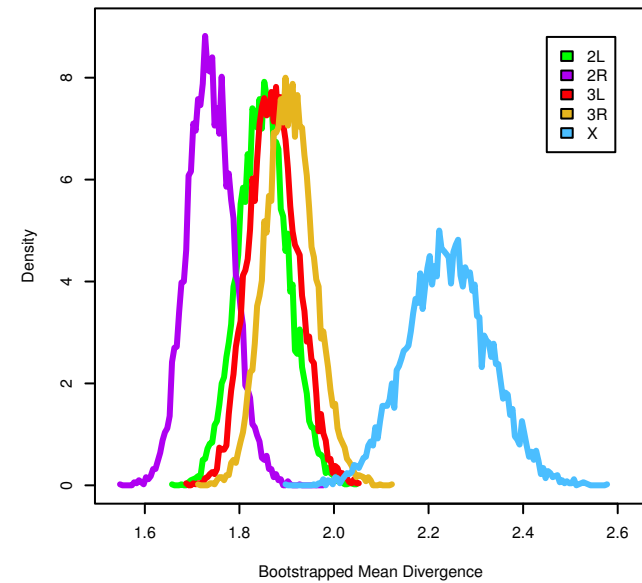

Supplement: Figure S24 — Gene expression divergence on the X chromosome relative to the autosomes for sets of genes with groups of non-expressed genes removed using various different criteria: non-expressed in at least two species in at least one time point (“Two”), non-expressed in at least six species in at least one time point (“Six”), and non-expressed in all species at all time points (“Six-Eight”). See Methods. (PDF) [file pgen.1003200.s024.pdf]

$$\rho_{\text{TOTAL}} = -0.42$$

$$\rho_{\text{A}} = 0.50$$

$$\rho_{\text{B}} = 0.66$$

$$\rho_{\text{A}:\text{B}} = -0.96$$

$$\rho_{\text{B}:\text{A}} = -0.95$$

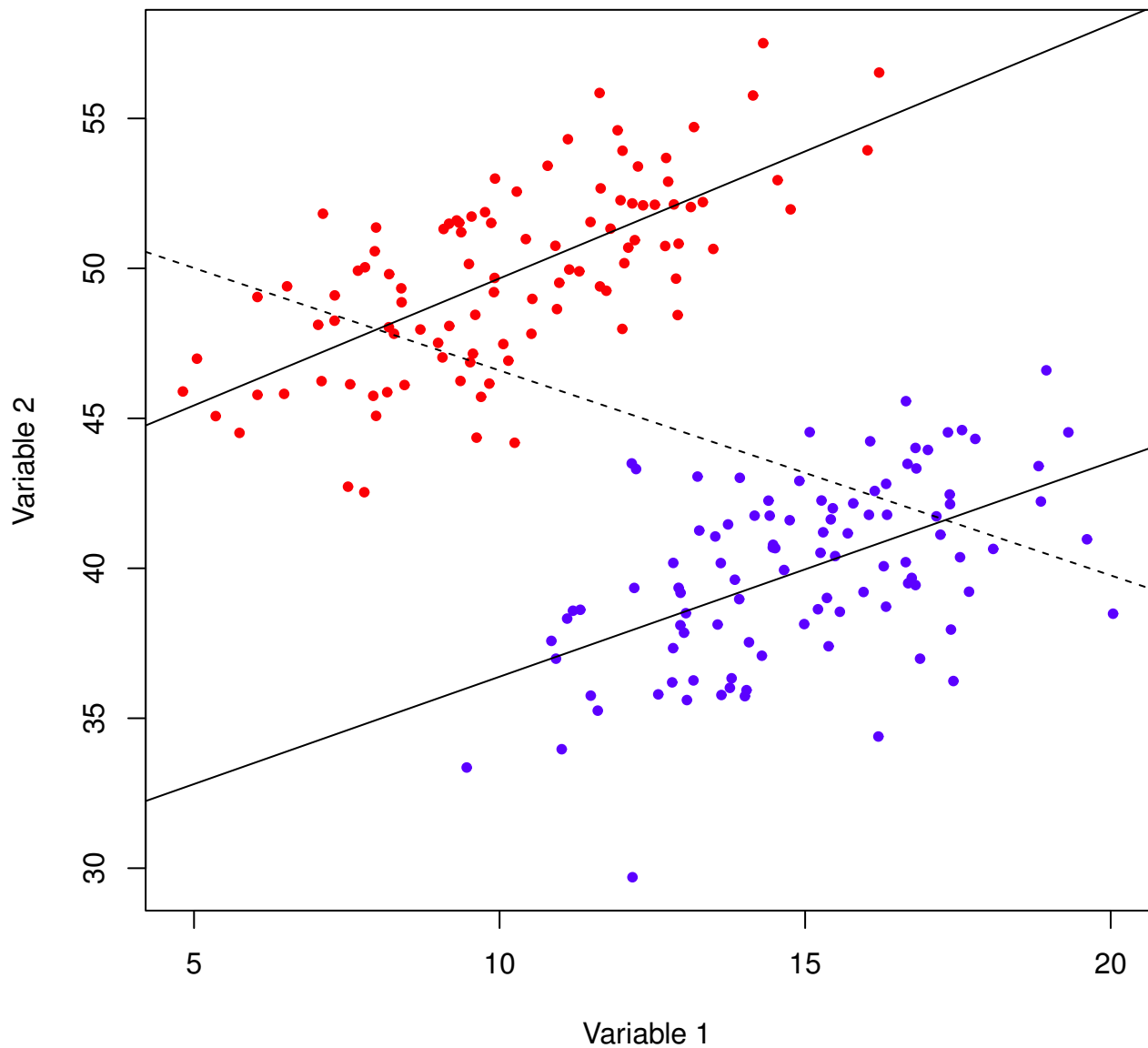

Supplement: Figure S25 — Simulated bivariate data illustrating the Yule-Simpson effect [91]–[95] when correlating subsets that belong to a larger aggregate. The red and blue points represent two subsets within the total population which display positive correlations when correlated as subsets (unbroken lines) yet a negative correlation when taken as a total population (dashed line). When we use a relativised Spearman's correlation (see Methods), however, we find that these subsets display negative correlations relative to each other thereby explaining why there is a negative correlation for the total population. (PDF) [file pgen.1003200.s025.pdf]

Embryos

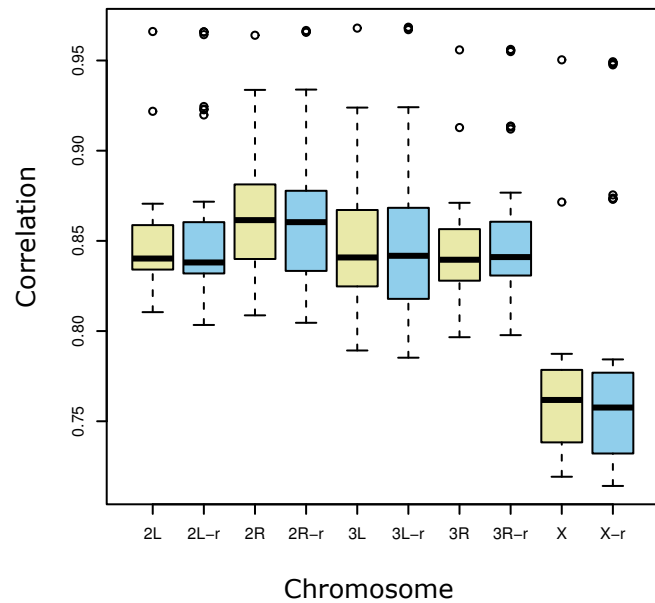

Adult Males

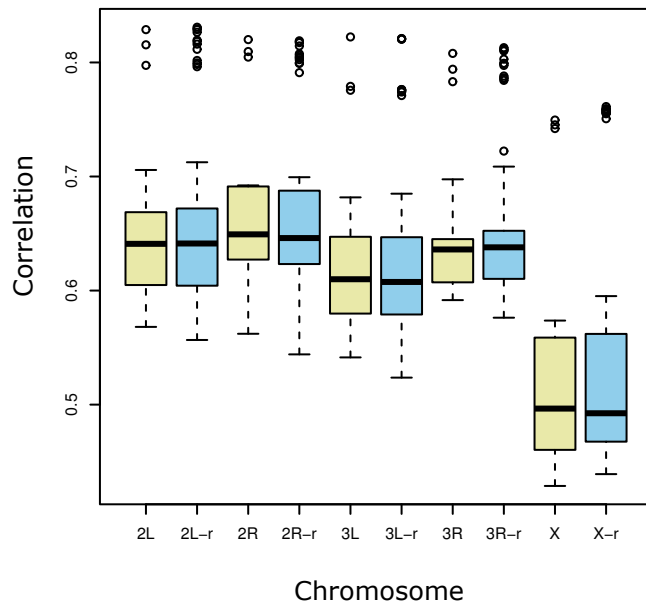

Adult Females

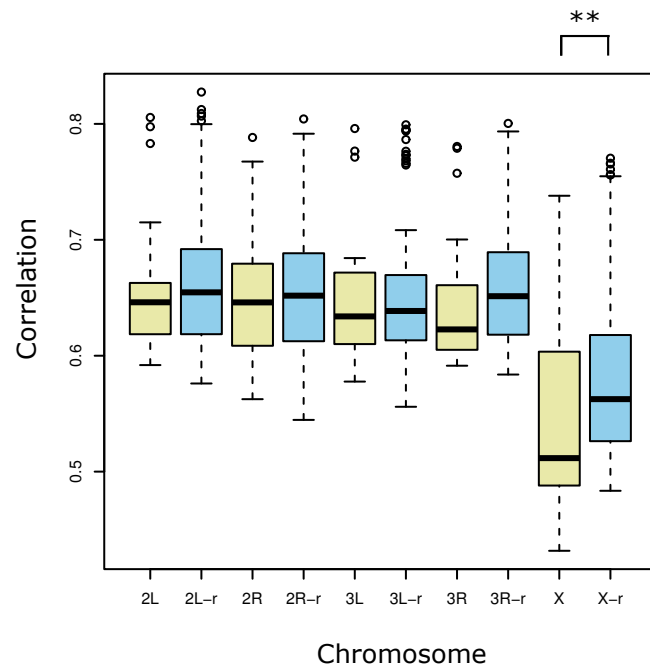

Supplement: Figure S26 — Distributions of pairwise species chromosome correlations for embryos, adult males, and adult females. In light blue are the distributions of a relativised Spearman's rank correlation coefficient (see Methods). The suffix “_r” indicates that these are the relative correlation coefficients for a particular chromosome in relation to the other chromosomes. (PDF) [file pgen.1003200.s026.pdf]
