# Supplementary material for: An Excess of Gene Expression Divergence on the X Chromosome in Drosophila Embryos: Implications for the Faster-X Hypothesis
Source: PLoS Genet. 2012 Dec 27;8(12):e1003200. doi: 10.1371/journal.pgen.1003200 (PMC3531489; doi:10.1371/journal.pgen.1003200)
Supplement: Table S1 — The chromosomal distribution of genes in the expression datasets. (PDF) [file pgen.1003200.s027.pdf]

Supplementary Table 1: **The chromosomal distribution of genes in the expression datasets**

Stage	Comparison	2L	2R	3L	3R	X	Total
Embryos	species	579	651	677	798	314	3019
Embryos	strains	2267	2521	2437	3005	1998	12228
Adults	species	1214	1360	1215	1662	881	6332
Adults	strains	1694	1843	1721	2217	1275	8750
