# Supplementary material for: An Excess of Gene Expression Divergence on the X Chromosome in Drosophila Embryos: Implications for the Faster-X Hypothesis
Source: PLoS Genet. 2012 Dec 27;8(12):e1003200. doi: 10.1371/journal.pgen.1003200 (PMC3531489; doi:10.1371/journal.pgen.1003200)
Supplement: Table S2 — Contrasts for Drosophila embryo species comparisons. Aut - all autosomes. W - Wilcoxon rank sum test statistic. P-values adjusted according to Benjamini-Hochberg correction. (PDF) [file pgen.1003200.s028.pdf]

Supplementary Table 2: **Contrasts for *Drosophila* embryo species comparisons**

Contrast	Mean 1st	Mean 2nd	W-stat	P-value	P_{adj}-value
Aut-X	1.912150	2.312695	331955.5	2.19×10^{-7}	-
2L-X	1.906623	2.312695	74430	3.8×10^{-6}	1.9×10^{-5}
2R-X	1.808078	2.312695	79352	8.8×10^{-9}	8.8×10^{-8}
3L-X	1.951095	2.312695	89006	1.8×10^{-5}	4.7×10^{-5}
3R-X	1.963467	2.312695	104232.5	6.3×10^{-6}	2.1×10^{-5}
2L-2R	1.906623	1.808078	196983.5	0.171	0.244
2L-3L	1.906623	1.951095	193720	0.723	0.803
2L-3R	1.906623	1.963467	227856	0.664	0.803
2R-3L	1.808078	1.951095	208468	0.089	0.148
2R-3R	1.808078	1.963467	244404	0.053	0.106
3L-3R	1.951095	1.963467	269368	0.926	0.926

Aut - all autosomes. W - Wilcoxon rank sum test statistic. P-values adjusted according to Benjamini-Hochberg correction.
