# Supplementary material for: An Excess of Gene Expression Divergence on the X Chromosome in Drosophila Embryos: Implications for the Faster-X Hypothesis
Source: PLoS Genet. 2012 Dec 27;8(12):e1003200. doi: 10.1371/journal.pgen.1003200 (PMC3531489; doi:10.1371/journal.pgen.1003200)
Supplement: Table S3 — Contrasts for D. melanogaster embryo strain comparisons. Aut - all autosomes. W - Wilcoxon rank sum test statistic. P-values adjusted according to Benjamini-Hochberg correction. (PDF) [file pgen.1003200.s029.pdf]

Supplementary Table 3: **Contrasts for *D. melanogaster* embryo strain comparisons**

Contrast	Mean 1st	Mean 2nd	W-stat	P-value	P_{adj}-value
Aut-X	1.400482	1.287024	11273798	1.16 x 10^{-9}	-
2L-X	1.411071	1.287024	2496381	7.8 x 10^{-9}	7.8 x 10^{-8}
2R-X	1.395688	1.287024	2731109	1.1 x 10^{-6}	3.5 x 10^{-6}
3L-X	1.422667	1.287024	2667918	3.8 x 10^{-9}	1.9 x 10^{-7}
3R-X	1.380755	1.287024	3205879	4.6 x 10^{-5}	1.2 x 10^{-4}
2L-2R	1.411071	1.395688	2905198	0.318	0.424
2L-3L	1.411071	1.422667	2771018	0.852	0.852
2L-3R	1.411071	1.380755	3514421	0.048	0.096
2R-3L	1.395688	1.422667	3032346	0.433	0.481
2R-3R	1.395688	1.380755	3844265	0.339	0.424
3L-3R	1.422667	1.380755	3764470	0.074	0.123

Aut - all autosomes. W - Wilcoxon rank sum test statistic. P-values adjusted according to Benjamini-Hochberg correction.
