# Supplementary material for: An Excess of Gene Expression Divergence on the X Chromosome in Drosophila Embryos: Implications for the Faster-X Hypothesis
Source: PLoS Genet. 2012 Dec 27;8(12):e1003200. doi: 10.1371/journal.pgen.1003200 (PMC3531489; doi:10.1371/journal.pgen.1003200)
Supplement: Table S4 — Contrasts for Drosophila adult female species comparisons. Aut - all autosomes. W - Wilcoxon rank sum test statistic. P-values adjusted according to Benjamini-Hochberg correction. (PDF) [file pgen.1003200.s030.pdf]

Supplementary Table 4: **Contrasts for *Drosophila* adult female species comparisons**

Contrast	Mean 1st	Mean 2nd	W-stat	P-value	P_{adj}-value
Aut-X	1.128154	1.129415	2409026	0.994	-
2L-X	1.118199	1.129415	528305.5	0.636	-
2R-X	1.127064	1.129415	599021	0.997	-
3L-X	1.141319	1.129415	543405.5	0.549	-
3R-X	1.126056	1.129415	729981	0.904	-
2L-2R	1.118199	1.127064	815877.5	0.609	-
2L-3L	1.118199	1.141319	717046	0.237	-
2L-3R	1.118199	1.126056	999595.5	0.675	-
2R-3L	1.127064	1.141319	812860.5	0.479	-
2R-3R	1.127064	1.126056	1133633	0.884	-
3L-3R	1.141319	1.126056	1028575	0.390	-

Aut - all autosomes. W - Wilcoxon rank sum test statistic. P-values adjusted according to Benjamini-Hochberg correction.
