# Supplementary material for: An Excess of Gene Expression Divergence on the X Chromosome in Drosophila Embryos: Implications for the Faster-X Hypothesis
Source: PLoS Genet. 2012 Dec 27;8(12):e1003200. doi: 10.1371/journal.pgen.1003200 (PMC3531489; doi:10.1371/journal.pgen.1003200)
Supplement: Table S5 — Contrasts for D. melanogaster female adult strain comparisons. Aut - all autosomes. W - Wilcoxon rank sum test statistic. P-values adjusted according to Benjamini-Hochberg correction. (PDF) [file pgen.1003200.s031.pdf]

Supplementary Table 5: **Contrasts for *Drosophila* adult male species comparisons**

Contrast	Mean 1st	Mean 2nd	W-stat	P-value	P_{adj}-value
Aut-X	1.168791	1.184388	2361976	0.3552	-
2L-X	1.170921	1.184388	525043.5	0.477	-
2R-X	1.159897	1.184388	584673	0.336	-
3L-X	1.172074	1.184388	525263	0.467	-
3R-X	1.169593	1.184388	717711.5	0.413	-
2L-2R	1.170921	1.159897	830314	0.800	-
2L-3L	1.170921	1.172074	738058	0.975	-
2L-3R	1.170921	1.169593	1011397	0.907	-
2R-3L	1.159897	1.172074	822211.5	0.832	-
2R-3R	1.159897	1.169593	1125957	0.860	-
3L-3R	1.1195	1.169593	1011382	0.938	-

Aut - all autosomes. W - Wilcoxon rank sum test statistic. P-values adjusted according to Benjamini-Hochberg correction.
