# Supplementary material for: An Excess of Gene Expression Divergence on the X Chromosome in Drosophila Embryos: Implications for the Faster-X Hypothesis
Source: PLoS Genet. 2012 Dec 27;8(12):e1003200. doi: 10.1371/journal.pgen.1003200 (PMC3531489; doi:10.1371/journal.pgen.1003200)
Supplement: Table S6 — Contrasts for Drosophila adult male species comparisons. Aut - all autosomes. W - Wilcoxon rank sum test statistic. P-values adjusted according to Benjamini-Hochberg correction. (PDF) [file pgen.1003200.s032.pdf]

Supplementary Table 6: **Contrasts for *D. melanogaster* female adult strain comparisons**

Contrast	Mean 1st	Mean 2nd	W-stat	P-value	P_{adj}-value
Aut-X	0.6113846	0.5431882	5192587	7.28 x 10^{-6}	-
2L-X	0.6399925	0.5431882	1219771	1.46 x 10^{-9}	1.68 x 10^{-8}
2R-X	0.6033502	0.5431882	1244176	0.0051	0.0078
3L-X	0.6187143	0.5431882	1189794	3.78 x 10^{-5}	0.00020
3R-X	0.5943979	0.5431882	1494048	0.0025	0.0046
2L-2R	0.6399925	0.6033502	1663789	7.1 x 10^{-4}	0.0046
2L-3L	0.6399925	0.6187143	1520440	0.029	0.040
2L-3R	0.6399925	0.5943979	2010076	1.6 x 10^{-4}	0.0019
2R-3L	0.6033502	0.6187143	1548129	0.219	0.240
2R-3R	0.6033502	0.5943979	2049155	0.868	0.868
3L-3R	0.6187143	0.5943979	1959594	0.143	0.175

Aut - all autosomes. W - Wilcoxon rank sum test statistic. P-values adjusted according to Benjamini-Hochberg correction.
