# Supplementary material for: An Excess of Gene Expression Divergence on the X Chromosome in Drosophila Embryos: Implications for the Faster-X Hypothesis
Source: PLoS Genet. 2012 Dec 27;8(12):e1003200. doi: 10.1371/journal.pgen.1003200 (PMC3531489; doi:10.1371/journal.pgen.1003200)
Supplement: Table S7 — Contrasts for D. melanogaster male adult strain comparisons. Aut - all autosomes. W - Wilcoxon rank sum test statistic. P-values adjusted according to Benjamini-Hochberg correction. (PDF) [file pgen.1003200.s033.pdf]

Supplementary Table 7: **Contrasts for *D. melanogaster* male adult strain comparisons**

Contrast	Mean 1st	Mean 2nd	W-stat	P-value	P_{adj}-value
Aut-X	0.4768255	0.4189356	5359535	9.89×10^{-11}	-
2L-X	0.4877695	0.4189356	1249111	2.52×10^{-13}	2.56×10^{-12}
2R-X	0.4705672	0.4189356	1281926	1.49×10^{-5}	4.97×10^{-5}
3L-X	0.4884414	0.4189356	1255394	1.38×10^{-11}	6.89×10^{-11}
3R-X	0.467385	0.4189356	1526993	7.42×10^{-5}	1.86×10^{-4}
2L-2R	0.4877695	0.4705672	1657322	0.0015	0.0021
2L-3L	0.4877695	0.4884414	1473490	0.583	0.583
2L-3R	0.4877695	0.467385	2013942	9.99×10^{-5}	2.00×10^{-4}
2R-3L	0.4705672	0.4884414	1505481	0.0088	0.011
2R-3R	0.4705672	0.467385	2064474	0.563	0.583
3L-3R	0.4884414	0.467385	2025011	9.20×10^{-4}	0.0015

Aut - all autosomes. W - Wilcoxon rank sum test statistic. P-values adjusted according to Benjamini-Hochberg correction.
