# Supplementary material for: An Excess of Gene Expression Divergence on the X Chromosome in Drosophila Embryos: Implications for the Faster-X Hypothesis
Source: PLoS Genet. 2012 Dec 27;8(12):e1003200. doi: 10.1371/journal.pgen.1003200 (PMC3531489; doi:10.1371/journal.pgen.1003200)
Supplement: Table S8 — Contrasts for Drosophila embryos for a common set of 2072 genes and 5 species. W - Wilcoxon rank sum test statistic. P-values adjusted according to Benjamini-Hochberg correction. (PDF) [file pgen.1003200.s034.pdf]

Supplementary Table 8: **Contrasts for *Drosophila* embryos for a common set of 2072 genes and 5 species.**

Contrast	Mean 1st	Mean 2nd	W-stat	P-value	P_{adj}-value
2L-X	1.767753	2.023779	39029	0.009315	0.0232
2R-X	1.647628	2.023779	39580	5.4 x 10^{-5}	5.4 x 10^{-4}
3L-X	1.759429	2.023779	40418	0.004624	0.0154
3R-X	1.736327	2.023779	51914.5	8.5 x 10^{-4}	0.0042
2L-2R	1.767753	1.647628	99783	0.02553	0.051
2L-3L	1.767753	1.759429	89248	0.3774	0.377
2L-3R	1.767753	1.736327	119319	0.2105	0.263
2R-3L	1.647628	1.759429	91380	0.06257	0.104
2R-3R	1.647628	1.736327	121461.5	0.09044	0.129
3L-3R	1.759429	1.736327	123275	0.3443	0.377

W - Wilcoxon rank sum test statistic. P-values adjusted according to Benjamini-Hochberg correction.
