# Supplementary material for: An Excess of Gene Expression Divergence on the X Chromosome in Drosophila Embryos: Implications for the Faster-X Hypothesis
Source: PLoS Genet. 2012 Dec 27;8(12):e1003200. doi: 10.1371/journal.pgen.1003200 (PMC3531489; doi:10.1371/journal.pgen.1003200)
Supplement: Table S9 — Contrasts for Drosophila adults for a common set of 2072 genes and 5 species. W - Wilcoxon rank sum test statistic. P-values adjusted according to Benjamini-Hochberg correction. (PDF) [file pgen.1003200.s035.pdf]

Supplementary Table 9: **Contrasts for *Drosophila* adults for a common set of 2072 genes and 5 species.**

Contrast	Mean 1st	Mean 2nd	W-stat	P-value	P_{adj}-value
2L-X	0.9187558	0.9741846	40504.5	0.04798	0.164
2R-X	0.90749	0.9741846	44751.5	0.04946	0.164
3L-X	0.9049189	0.9741846	41350	0.01444	0.144
3R-X	0.9461049	0.9741846	57753.5	0.1443	0.288
2L-2R	0.9187558	0.90749	92572	0.4904	0.490
2L-3L	0.9187558	0.9049189	90367.5	0.2641	0.293
2L-3R	0.9187558	0.9461049	112693	0.2353	0.293
2R-3L	0.90749	0.9049189	99597	0.2614	0.293
2R-3R	0.90749	0.9461049	124054	0.2174	0.293
3L-3R	0.9049189	0.9461049	115402	0.08814	0.220

W - Wilcoxon rank sum test statistic. P-values adjusted according to Benjamini-Hochberg correction.
