# Supplementary material for: An Excess of Gene Expression Divergence on the X Chromosome in Drosophila Embryos: Implications for the Faster-X Hypothesis
Source: PLoS Genet. 2012 Dec 27;8(12):e1003200. doi: 10.1371/journal.pgen.1003200 (PMC3531489; doi:10.1371/journal.pgen.1003200)
Supplement: Table S10 — Characterisation of the embryonic expression patterns of genes residing on the X chromosome in Drosophila. Enrichment is based on the ‘parent-child’ algorithm in the topGO R package and Fisher's exact test applied to 2228 genes that reside on the X chromosome in Drosophila, and enrichment is relative to the whole genome. Terms with uncorrected P-values below 0.05 are shown. # - total number of genes with this annotation in the dataset. Sig. - significant, Exp. - expected. -value - adjusted according to the Benjamini-Hochberg false discovery rate. (PDF) [file pgen.1003200.s036.pdf]

Supplementary Table 10: **Characterisation of genes with a percentile X/A divergence ratio greater than 1.015 in adult males.**

ID	Term	#	Sig.	Exp.	P-value	P_{adj}-value
GO:0007538	primary sex determination	11	5	0.6	8.9×10^{-5}	0.312
GO:0019748	secondary metabolic process	24	7	1.32	1.6×10^{-4}	0.312
GO:0030534	adult behavior	34	7	1.86	6.2×10^{-4}	0.625
GO:0007362	terminal region determination	10	3	0.5	0.0062	0.625
GO:0046152	ommochrome metabolic process	13	4	0.71	0.0076	1.0

Enrichment is based on the ‘parent-child’ algorithm in the topGO R package and Fisher’s exact test applied to 352 genes that have an X/A percentile divergence ratio of > 1.015 against the background of the genes in the dataset. # - total number of genes with this annotation in the dataset. Sig. - significant, Exp. - expected. P_{adj}-value - adjusted according to the Benjamini-Hochberg false discovery rate.
