# Supplementary material for: An Excess of Gene Expression Divergence on the X Chromosome in Drosophila Embryos: Implications for the Faster-X Hypothesis
Source: PLoS Genet. 2012 Dec 27;8(12):e1003200. doi: 10.1371/journal.pgen.1003200 (PMC3531489; doi:10.1371/journal.pgen.1003200)
Supplement: Table S11 — Fitnesses in a diploid two-locus epistatic model with X-linkage. Fitnesses of different male-female gametic combinations when both the loci are located on the X chromosome. T/t - trans-acting gene; C/c - cis-acting locus; 00 - indicates a male gamete carrying a Y chromosome; - selection coefficient; - dominance coefficient. (PDF) [file pgen.1003200.s037.pdf]

Supplementary Table 11: **Characterisation of the embryonic expression patterns of genes residing on the X chromosome in *Drosophila*.**

Test	ID	Term	#	Sig.	Exp.	P-value	P_{adj}-value
Under	254	cellular blastoderm	3039	418	477.37	2.6 x 10^{-7}	9.5 x 10^{-5}
	273	visual anlage	100	7	15.71	0.017	1.0
	222	visual primordium	88	6	13.82	0.022	1.0
Over	493	no staining (stage 5)	2773	471	435.58	0.00029	0.10643
	346	muscle system primordium	670	109	105.24	0.00392	0.71392
	580	apically cleared	74	19	11.62	0.01309	1.0

Enrichment is based on the 'parent-child' algorithm in the topGO R package and Fisher's exact test applied to 2228 genes that reside on the X chromosome in *Drosophila*, and enrichment is relative to the whole genome. Terms with uncorrected P-values below 0.05 are shown. # - total number of genes with this annotation in the dataset. Sig. - significant, Exp. - expected. P_{adj}-value - adjusted according to the Benjamini-Hochberg false discovery rate.
