# Supplementary material for: An Excess of Gene Expression Divergence on the X Chromosome in Drosophila Embryos: Implications for the Faster-X Hypothesis
Source: PLoS Genet. 2012 Dec 27;8(12):e1003200. doi: 10.1371/journal.pgen.1003200 (PMC3531489; doi:10.1371/journal.pgen.1003200)
Supplement: Table S12 — Characterisation of genes with a percentile X/A divergence ratio greater than 1.015 in adult males. Enrichment is based on the ‘parent-child’ algorithm in the topGO R package and Fisher's exact test applied to 352 genes that have an X/A percentile divergence ratio of 1.015 against the background of the genes in the dataset. # - total number of genes with this annotation in the dataset. Sig. - significant, Exp. - expected. -value - adjusted according to the Benjamini-Hochberg false discovery rate. (PDF) [file pgen.1003200.s038.pdf]

Supplementary Table 12: Fitnesses in a diploid two-locus epistatic model with X-linkage.

		♂				
		TC	Tc	tC	tc	00
♀	TC	1	1	1	$1+\frac{h}{2}s$	1
	Tc	1	1	$1+\frac{h}{2}s$	$1+hs$	1
	tC	1	$1+\frac{h}{2}s$	1	$1+hs$	1
	tc	$1+\frac{h}{2}s$	$1+hs$	$1+hs$	$1+s$	$1+s$

Fitnesses of different male-female gametic combinations when both the loci are located on the X chromosome. T/t - trans-acting gene; C/c - cis-acting locus; 00 - indicates a male gamete carrying a Y chromosome; s - selection coefficient; h - dominance coefficient.
