# Supplementary material for: An Excess of Gene Expression Divergence on the X Chromosome in Drosophila Embryos: Implications for the Faster-X Hypothesis
Source: PLoS Genet. 2012 Dec 27;8(12):e1003200. doi: 10.1371/journal.pgen.1003200 (PMC3531489; doi:10.1371/journal.pgen.1003200)
Supplement: Table S13 — Fitnesses in a diploid two-locus epistatic model. Fitnesses of different male-female gametic combinations when there is a beneficial partially recessive interaction between an autosomal allele and an X-linked allele (males are the heterogametic sex). T/t - trans-acting autosomal gene; C/c - cis-acting X-linked locus; 0 - indicates a male gamete carrying a Y chromosome; - selection coefficient; - dominance coefficient. (PDF) [file pgen.1003200.s039.pdf]

Supplementary Table 13: Fitnesses in a diploid two-locus epistatic model.

		♂					
		TC	Tc	tC	tc	T0	t0
♀	TC	1	1	1	$1+\frac{h}{2}s$	1	1
	Tc	1	1	$1+\frac{h}{2}s$	$1+hs$	1	$1+hs$
	tC	1	$1+\frac{h}{2}s$	1	$1+hs$	1	1
	tc	$1+\frac{h}{2}s$	$1+hs$	$1+hs$	$1+s$	$1+hs$	$1+s$

Fitnesses of different male-female gametic combinations when there is a beneficial partially recessive interaction between an autosomal allele and an X-linked allele (males are the heterogametic sex). T/t - trans-acting autosomal gene; C/c - cis-acting X-linked locus; 0 - indicates a male gamete carrying a Y chromosome; s - selection coefficient; h - dominance coefficient.
